# NEDD8-activating enzyme inhibition potentiates the anti-myeloma activity of natural killer cells

**DOI:** 10.1038/s41419-023-05949-z

**Published:** 2023-07-17

**Authors:** Sara Petillo, Elena Sproviero, Luisa Loconte, Lorenzo Cuollo, Alessandra Zingoni, Rosa Molfetta, Cinzia Fionda, Alessandra Soriani, Cristina Cerboni, Maria Teresa Petrucci, Francesca Fazio, Rossella Paolini, Angela Santoni, Marco Cippitelli

**Affiliations:** 1 Department of Molecular Medicine, Sapienza University of Rome, Rome, Italy; 2 Hematology, Department of Translational and Precision Medicine, Sapienza University of Rome, Rome, Italy; 3 Istituto Pasteur-Fondazione Cenci Bolognetti, Rome, Italy; 4 IRCCS, Neuromed, Pozzilli, Italy

**Keywords:** Tumour immunology, Myeloma

## Abstract

Natural Killer (NK) cells act as important regulators in the development and progression of hematological malignancies and their suppressor activity against Multiple Myeloma (MM) cells has been confirmed in many studies. Significant changes in the distribution of NK cell subsets and dysfunctions of NK cell effector activities were described in MM patients and correlated with disease staging. Thus, restoring or enhancing the functionality of these effectors for the treatment of MM represents a critical need. Neddylation is a post-translational modification that adds a ubiquitin-like molecule, NEDD8, to the substrate protein. One of the outcomes is the activation of the Cullin Ring Ligases (CRLs), a class of ubiquitin-ligases that controls the degradation of about 20% of proteasome-regulated proteins. Overactivation of CRLs has been described in cancer and can lead to tumor growth and progression. Thus, targeting neddylation represents an attractive approach for cancer treatment. Our group has recently described how pharmacologic inhibition of neddylation increases the expression of the NKG2D activating receptor ligands, MICA and MICB, in MM cells, making these cells more susceptible to NK cell degranulation and killing. Here, we extended our investigation to the direct role of neddylation on NK cell effector functions exerted against MM. We observed that inhibition of neddylation enhanced NK cell-mediated degranulation and killing against MM cells and improved Daratumumab/Elotuzumab-mediated response. Mechanistically, inhibition of neddylation increased the expression of Rac1 and RhoA GTPases in NK cells, critical mediators for an efficient degranulation at the immunological synapse of cytotoxic lymphocytes, and augmented the levels of F-actin and perforin polarization in NK cells contacting target cells. Moreover, inhibition of neddylation partially abrogated TGFβ-mediated repression of NK cell effector activity. This study describes the role of neddylation on NK cell effector functions and highlights the positive immunomodulatory effects achieved by the inhibition of this pathway in MM.

## Introduction

MM is the second most common hematological cancer in the world, with a higher incidence in high-income countries [1]. For decades the standard of care for MM patients has been represented by chemotherapy in combination with steroids and, for eligible patients, hematopoietic stem cell transplantation. A great advance has been done in the treatment of this cancer with the introduction of target therapies such as immunomodulatory drugs (IMiDs, e.g. Lenalidomide/Pomalidomide), proteasome inhibitors [(PIs) i.e. Bortezomib)] and monoclonal antibodies (e.g. Daratumumab and Elotuzumab). These treatments have demonstrated an increased efficacy with deeper responses and a longer median survival which now exceeds six to seven years. However, most patients become resistant, thus making MM still an incurable cancer [2–4].

An important determinant for MM progression and development of drug resistance is the interaction of MM cells with the highly immunosuppressive tumor microenvironment (TME) of the bone marrow (BM-TME) where the tumor grows. The BM niche fosters MM survival and progression protecting these cells from apoptotic stimuli, while effector immune cells become progressively anergic and unable to restrain tumor growth [5, 6]. TME includes a cellular compartment composed by immune and non-immune cells and a non-cellular compartment formed by the extracellular matrix and soluble factors such as cytokines, growth factors and chemokines [5]. Among the soluble factors, a potent regulator of the TME is TGFβ, secreted by MM cells, bone marrow stromal cells (BMSCs), osteoblasts and Tregs [7], which hinders at various levels the anti-myeloma activity of cytotoxic lymphocytes. In particular, for NK cells, it can downregulate both their natural and Antibody Dependent Cellular Cytotoxicity (ADCC) [8], the expression of activating receptors, such as NKp30 and NKG2D, as well as IFNγ production [9–13]. The importance of NK cells in the control of progression and the eradication of MM has been well recognized both in mice [14, 15] and in humans [16–18], as well as the impairment of their effector functions during MM progression [19–22]. In this context, a number of activating receptors are responsible for NK cell recognition and killing of MM cells, among which NKG2D, DNAM-1 and the NCRs (NKp46, NKp30, NKp44) have particular relevance [17, 23, 24]. However, to avoid recognition MM cells can undergo decreased surface expression of NK cell-activating ligands (e.g. NKG2DLs) [24, 25], while expressing, together with other cell populations in the BM, ligands for inhibitory receptors such as the ligand for PD-1 (PD-L1) [22, 26, 27], providing mechanisms of tumor escape. In this scenario, target therapies able to restore or increase NK cell reactivity toward MM cells, in addition to their direct anti-MM activity, could be promising to gain a complete and long-lasting response.

Neddylation is a post-translational modification that adds a ubiquitin-like protein, NEDD8 (neuronal precursor cell-expressed developmentally downregulated protein 8), to selected substrates affecting their stability, subcellular localization, and conformation/function. As for ubiquitin, NEDD8 is prior activated and then conjugated to the target protein by a highly regulated enzymatic cascade involving E1 (NAE), E2 (UBE2M and UBE2F) and E3 (e.g. RBX1/2) enzymes. Neddylation regulates many biological and pathological processes, including cancer progression and immune response [28–31]. In the past decade, many regulators of the Neddylation pathway have been identified. MLN4924 (Pevonedistat) is a first-in-class inhibitor which prevents the conjugation of NEDD8 by blocking the activity of the only NEDD8 activating enzyme NAE. MLN4924 is currently involved in phase I/II/III clinical trials for patients suffering from solid and hematological malignancies, including MM [32–37]. Neddylation inhibition has been shown to exert several anti‐tumor activities by modulating critical cellular processes in tumor cells [28, 38, 39], however the effects of reduced neddylation on immune cell functions and in the context of the TME have not been well investigated yet. Recently, we described how neddylation inhibition can regulate the expression of NK cell activating ligands in MM cells and sensitize these cells to NK cell recognition and killing [40]. The functional implication of this upregulation is an increased NK cell activation, with a mechanism dependent on the receptor NKG2D, which leads to an enhanced degranulation and NK cell-mediated killing of MM target cells.

Here, we investigated the possible direct effects of neddylation inhibition on NK cells and on their anti-MM activity. Our data indicate that MLN4924/Pevonedistat, used in the nanomolar range of concentration, upregulates NK cell-mediated degranulation and killing towards MM cells; moreover, it potentiates Daratumumab and Elotuzumab-mediated NK cell degranulation. Mechanistically, neddylation inhibition significantly increases the expression of Rac1 and RhoA GTPases in NK cells, critical mediators for an efficient degranulation at the immunological synapse (IS) of cytotoxic lymphocytes [41–44], and augments the levels of F-actin and the efficiency of perforin polarization in NK cells contacting target cells.

Noteworthy, neddylation inhibition partially abrogated TGFβ-mediated repression of NK cell effector function and counteracted the suppressive activity of BM-derived plasma from MM patients, where TGFβ plays a major inhibitory role.

In conclusion, these findings provide new insights on the immuno-mediated antitumor activities of neddylation inhibition, by pointing out its direct effects on NK cell effector functions, and further elucidating the molecular mechanisms that regulate NK cell activity in the BM immunosuppressive TME of MM.

## Results

### Inhibition of neddylation enhances NK cell-mediated degranulation and killing against MM cells

**Fig. 1:**
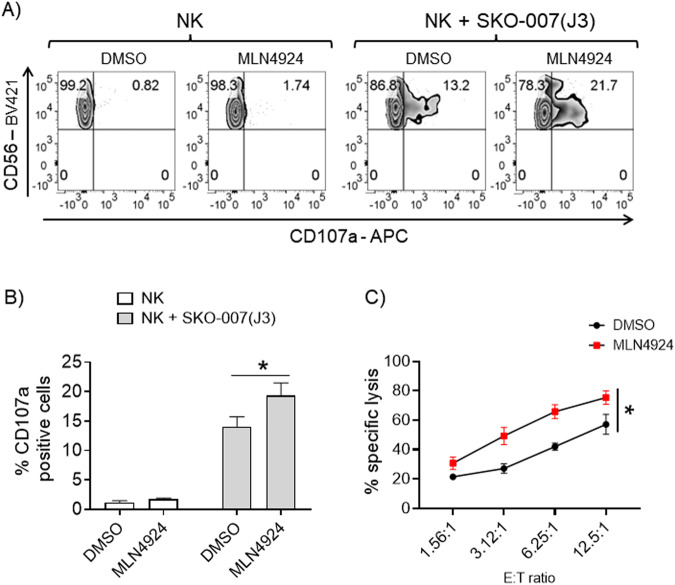
MLN4924 increases NK cell degranulation and cytotoxicity against SKO-007(J3) MM cells. **A** NK cell degranulation was evaluated using the lysosomal marker CD107a. As source of effector cells, we used PBMCs purified from healthy donor blood by Ficoll–Hypaque centrifugation. Cells, untreated or treated with MLN4924 (150 nM) for 72 h, were co-cultured with the SKO-007(J3) cell line used as target. The assay was performed at the Effector/Target (E/T) ratio of 2.5:1 in complete medium at 37 °C and 5% CO_2_ for 2 h. Cells were then stained with Fixable Viability Stain 780, anti-CD14-APC-H7, anti-CD19-APC-H7, anti-CD45-PE-Cy™7, anti-CD3-BV510, anti-CD138-PE, anti-CD56-BV421, anti-CD16-PerCP-Cy™5.5 and anti-CD107a-APC. CD107a expression was evaluated on NK cells gated as CD14^−^CD19^−^CD45^+^CD3^−^CD138^−^CD56^+^ using a FACS Canto II flow cytometer (BD Biosciences) and data were analyzed by FlowJo V10 Cytometric Analysis Software (BD Biosciences). A representative experiment is shown. **B** Percentage of CD107a positive cells represents the average of six independent experiments and statistical significance was evaluated by paired Student’s *t* test (*P < 0.05). **C** Cultivated NK cells from healthy donors, untreated or treated with MLN4924 (150 nM) for 48 h, were incubated with CFSE-labeled SKO-007(J3) cells used as target in 7-AAD/CFSE cell-mediated cytotoxicity assays. These assays were performed at E/T ratios ranging from 1.56:1 to 12.5:1. Percentage of specific lysis is reported. Results represent the average of three independent experiments (*P < 0.05).

**Fig. 2:**
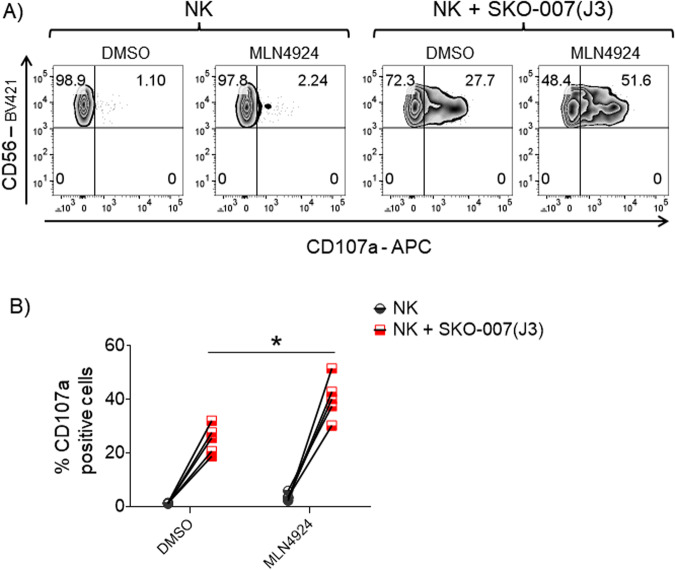
Patient-derived NK cells treated with MLN4924 show increased degranulation against SKO-007(J3) cells. **A** BMMCs depleted of MM cells, indicated as CD138^−^ cells, cultured for 48 h in complete medium supplemented with IL-2 (200 U/mL) and stimulated with MLN4924 (150 nM) or vehicle, were incubated with SKO-007(J3) cells used as target cells in a degranulation assay. The assay was performed at the E/T ratio of 2.5:1. After 2 h at 37 °C, cell surface expression of CD107a was analyzed on CD14^−^CD19^−^CD45^+^CD3^−^CD138^−^CD56^+^ NK cells, as described before. A representative experiment is shown. **B** The percentage of CD107a positive NK cells was reported for five independent experiments. Statistical significance was evaluated by paired Student’s *t* test (*P < 0.05).

We investigated the possible effects of neddylation inhibition on NK cell-mediated cytotoxicity. Using fresh isolated PBMCs from healthy donors, untreated or treated with the drug MLN4924 (150 nM) for 72 h, we analyzed by flow cytometry the cell surface expression of the lysosomal marker CD107a (a surrogate marker for cytotoxic granule exocytosis) on gated NK cells (Supplementary Fig. 1) upon interaction with different human MM cell lines [SKO-007(J3), ARK, RPMI 8226]. As shown in Fig. 1A, B (and Supplementary Fig. 2), basal expression of CD107a on NK cells increased when PBMCs were co-cultured with human MM target cells; this effect was further potentiated by MLN4924. These observations were also confirmed using 7-AAD/CFSE cell-mediated cytotoxicity assays, where SKO-007(J3) cells were lysed more efficiently by MLN4924-treated cultivated NK cells as compared to untreated controls (Fig. 1C). Accordingly, a higher degranulation capability was also observed in MLN4924-treated patient-derived NK cells gated on the CD138^-^ BM aspirate fraction (Supplementary Fig. 3), against SKO-007(J3) (Fig. 2A, B), ARK and RPMI 8226 cells (Supplementary Fig. 4). These treatments did not significantly affect cell viability at the concentration of MLN4924 (150 nM) and for the experimental time (48 or 72 h) chosen for these experiments (as assessed by Fixable Viability/780 staining, data not shown). Increased degranulation activity was also observed using the human NK cell line NK92, where the expression of the NAE heterodimeric enzyme subunits, NAE1/APPBP1 and UBA3, was reduced by lentiviral-mediated shRNA interference (Supplementary Fig. 5). Altogether, these data indicate that inhibition of neddylation increases NK cell degranulation and killing against MM cells.

### Antibody-mediated NK cell degranulation is improved by neddylation inhibition

**Fig. 3:**
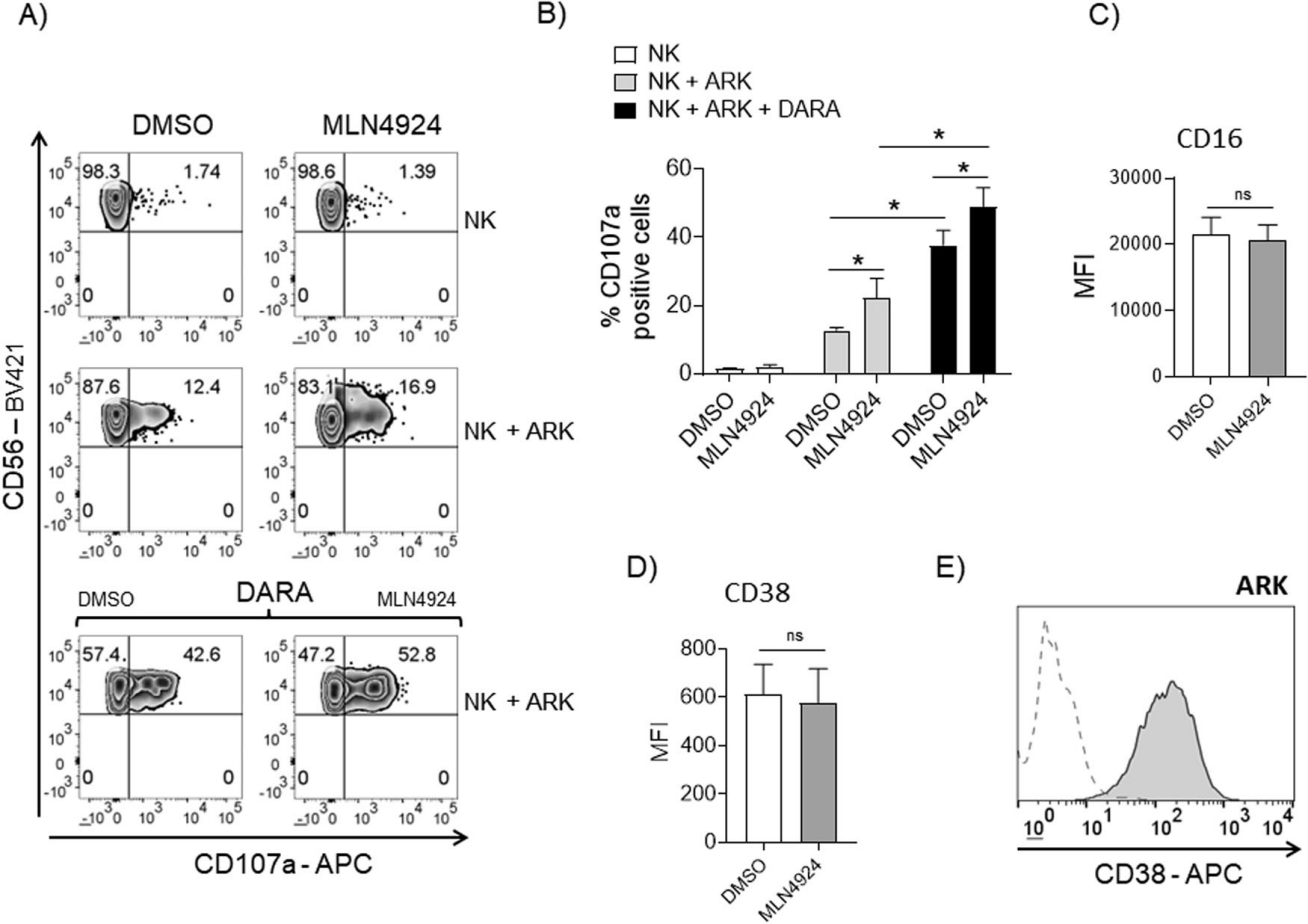
Daratumumab-mediated degranulation against MM cells is increased by Neddylation inhibition. **A** NK cell degranulation was evaluated using CD107a as previously described. As source of effector cells, we used PBMCs stimulated with MLN4924 (150 nM) or vehicle for 72 h, and incubated with ARK cells, pretreated or not with Daratumumab (DARA). Cells were stained with Fixable Viability Stain 780, anti-CD14-APC-H7, anti-CD19-APC-H7, anti-CD45-PE-Cy™7, anti-CD3-BV510, anti-CD138-PE, anti-CD56-BV421, anti-CD16-PerCP-Cy™5.5 and anti-CD107a-APC, and CD107a expression was analyzed using a FACSCanto II flow cytometer (BD Biosciences) on CD14^−^CD19^−^CD45^+^CD3^−^CD138^−^CD56^+^ NK cells. Data were analyzed by FlowJo V10 Cytometric Analysis Software (BD Biosciences). A representative experiment is shown. **B** Average percentage of CD107a positive cells was calculated based on three independent experiments and statistical significance was evaluated by ANOVA (*P < 0.05). Expression of CD16 (**C**) and CD38 (**D**) on NK cells gated on PBMCs, as described before, untreated or treated with MLN4924 (150 nM) for 72 h. Histograms represent the average MFI of four independent experiments. Statistical significance was evaluated by paired Student’s *t* test (*P < 0.05). **E** Expression of CD38 on ARK MM cells.

**Fig. 4:**
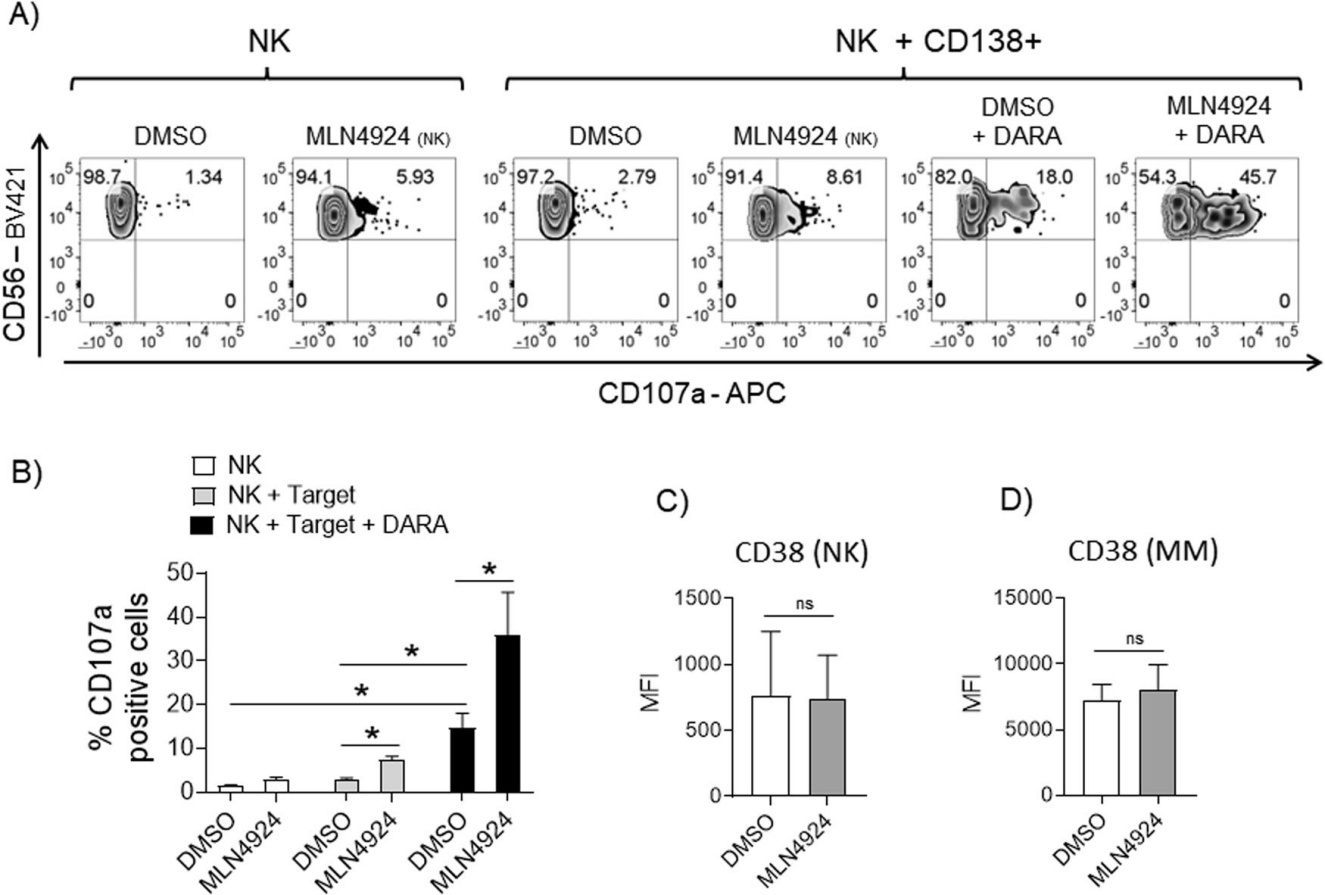
Higher Daratumumab-mediated degranulation in MLN4924-treated patient-derived NK cells against autologous MM target cells. **A** CD138^−^ bone marrow cells were left untreated or treated with MLN4924 (150 nM) for 48 h in complete medium supplemented with IL-2 (200 U/mL), and incubated with purified autologous MM cells, used as targets in degranulation assays. In some cases, MM cells were pre-treated with Daratumumab (1 µg/10^6^ cells) for 15 min at RT, washed twice and used as targets. The assay was performed at the E/T ratio of 2.5:1. After 2 h at 37 °C, cell surface expression of CD107a was analyzed on CD14^−^CD19^−^CD45^+^CD3^−^CD138^−^CD56^+^ cells. A representative degranulation assay is reported. **B** Histograms represent the average percentage of CD107a positive NK cells obtained from four patients (*P < 0.05). **C** Expression of CD38 was evaluated on NK cells from the CD138^−^ fraction (CD14^−^CD19^−^CD45^+^CD3^−^CD138^−^CD56^+^ cells) and **D** Expression of CD38 was evaluated on MM cells (CD14^−^CD19^−^CD45^+^CD3^−^CD138^+^ cells) treated with MLN4924 (150 nM) or left untreated for 48 h. Histograms represent the average MFI of three independent experiments and statistical significance was evaluated by paired Student’s *t* test (*P < 0.05).

Antibody-Dependent Cellular Cytotoxicity (ADCC) is a powerful cytolytic mechanism mediated by NK cells and at the basis of the clinical efficacy of many therapeutic monoclonal antibodies [45]. In this regard, any clinical strategy able to improve its efficacy represents a primary goal in onco-immunology. Daratumumab is an anti-CD38 monoclonal antibody that directs Complement-Dependent Cytotoxicity (CDC), Antibody-Dependent Cellular Phagocytosis (ADCP) as well as ADCC of CD38^+^ MM cells mediated by NK cells. A main interest of our study was to understand whether modulation of neddylation could interfere with the capability of NK cells to perform ADCC against MM cells. To clarify this point, we set up degranulation assays using fresh isolated PBMCs (untreated or treated with MLN4924) as effector cells, against the CD38^+^ MM cell line ARK (Fig. 3E). As shown in Fig. 3A, B, plasma membrane expression of CD107a on gated NK cells was strongly increased when ARK cells were pre-treated with Daratumumab, and this effect was further enhanced in MLN4924-treated PBMCs. In these experiments MLN4924 did not modulate the expression of CD16 on NK cells or the expression of CD38 on ARK cells (Fig. 3C, D). Accordingly, a higher degranulation was also observed for MLN4924-treated patient-derived NK cells, gated on the CD138^−^ BM aspirate fraction, against ARK cells that have prior experienced Daratumumab (Supplementary Fig. 6). To confirm these data in an effector/target autologous setting, we carried out degranulation assays using as effectors CD138^-^ cells isolated from BM aspirates of MM patients, untreated or treated with MLN4924 (150 nM) for 48 h and incubated with purified autologous myeloma target cells (CD138^+^ cells). In this setting, basal plasma membrane expression of CD107a on gated NK cells was increased by autologous MM target cells and MLN4924. Noteworthy, MLN4924 treatment further increased Daratumumab-induced degranulation, indicating that inhibition of neddylation is not detrimental but rather potentiates mAb/CD16-mediated degranulation against autologous MM cells (Fig. 4A, B). In these experiments MLN4924 did not modulate the expression of CD38 on NK or on MM cells as shown in Fig. 4C, D. We also extended these observations using Elotuzumab, a monoclonal antibody that targets signaling lymphocyte activation molecule family member 7 (SLAMF7/CS1) expressed on the surface of MM and NK cells, and shown to have significant clinical activity in combination therapies for relapsed/refractory MM [46]. As shown in Supplementary Fig. 7A, B, membrane expression of CD107a on gated NK cells from PBMCs, was increased when SLAMF7^+^ARK cells were exposed to Elotuzumab, and this effect was further enhanced when PBMCs were treated with MLN4924. In these experiments MLN4924 did not modulate the expression of SLAMF7 on NK cells (Supplementary Fig. 7C). Accordingly, a higher degranulation was also confirmed for MLN4924-treated patient-derived NK cells (gated on CD138^−^ BM aspirate fraction), against ARK cells exposed to Elotuzumab (Supplementary Fig. 8). Altogether, these data indicate that inhibition of neddylation potentiates Daratumumab/Elotuzumab-mediated degranulation of NK cells against MM cells.

### Analysis of NK cell receptors and effector molecules following MLN4924 treatment

**Fig. 5:**
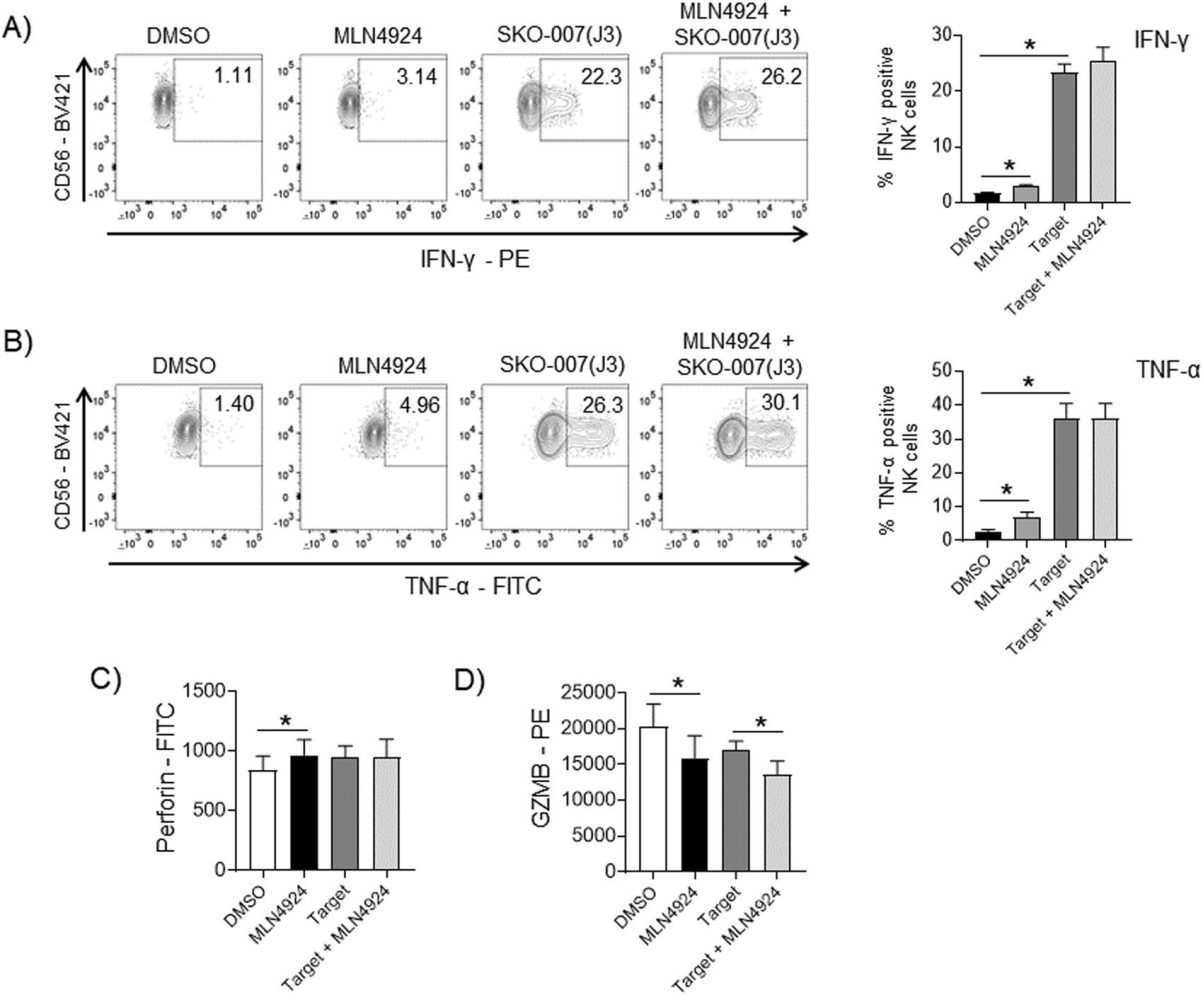
Analysis of NK cell effector molecules in MLN4924-treated NK cells. **A**, **B** Induction of IFN-γ and TNF-α in untreated or MLN4924 (150 nM)/72 h treated PBMCs contacting SKO-007(J3) target cells for 6 h. A representative graph and the average percentage of five experiments are shown. The expression of the indicated cytokines was analyzed on CD14^−^CD19^−^CD45^+^CD3^−^CD56^+^ NK cells. **C**, **D** Expression of Perforin and Granzyme B was analyzed as indicated above. Histograms represent the results of three independent experiments. Statistical significance was evaluated by paired Student’s *t* test (*P < 0.05). Fluorescence was analyzed using a FACS Canto II flow cytometer (BD Biosciences) and data were analyzed by FlowJo V10 Cytometric Analysis Software (BD Biosciences).

Previous observations from other groups showed that exposure of T cells to MLN4924 can regulate the production of different cytokines and affect activation and polarization of T-cell subsets [47–49]. We investigated the possible effects of MLN4924 on basal and target-induced production of IFN-γ and TNF-α in NK cells. As shown in Fig. 5A, B, MLN4924 did not significantly affect basal and target-induced levels of these two cytokines, as analyzed by flow cytometry on NK cells from PBMCs. In the same experimental setting, intracellular levels of Perforin were not significantly affected as well (Fig. 5C), while we observed a slight downregulation of Granzyme B levels (Fig. 5D). We also checked for the possible modulation by MLN4924 of several activating and inhibitory receptors mostly involved in NK cell-mediated immunosurveillance. As shown in Supplementary Fig. 9, MLN4924 slightly upregulated the expression of NKp30, NKp46 and PD1 in NK cells from PBMCs, while no significant changes were observed for NKG2D, DNAM1 and TIM3. A different picture was obtained for cultivated NK cells, where again NKp30 expression was slightly upregulated together with NKG2D and TIM3, while DNAM1 and PD1 were not affected and NKp46 was slightly downregulated (Supplementary Fig. 10). Altogether, these data indicate that MLN4924-induced higher cytotoxic potential of NK cells does not rely on increased levels of cytotoxic effector molecules, lytic granules, cytokine production, or on a significant reshaping of the activating/inhibitory receptor repertoire on the plasma membrane.

### MLN4924-induced enhancement of NK cell degranulation against MM: analysis of the molecular mechanisms involved

**Fig. 6:**
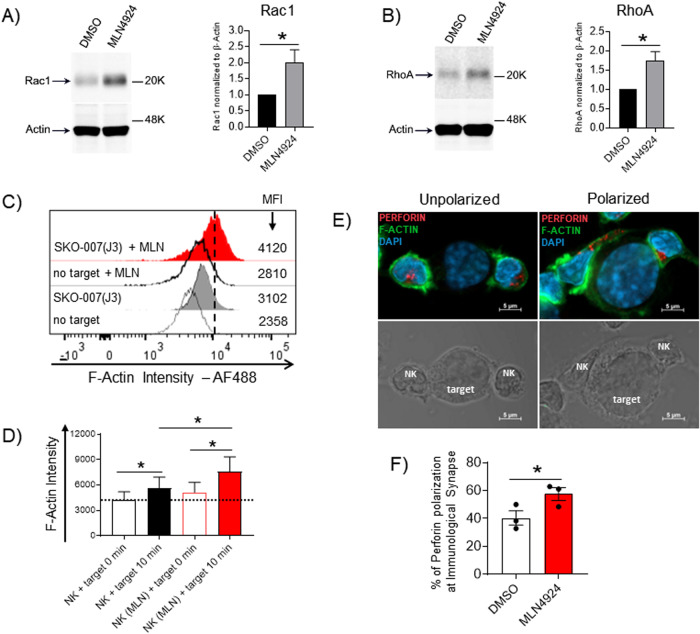
Expression of Rac1 and RhoA in cultivated NK cells exposed to MLN4924. Western blot analysis of Rac1 (**A**) and RhoA (**B**) in cultivated NK cells untreated or treated with MLN4924 (150 nM) for 48 h. β-Actin was used as protein loading control. A representative western blot is shown coupled to densitometric analysis, normalized to β-Actin, of three independent experiments. **C**, **D** Expression levels of F-actin were evaluated on cultivated NK cells treated with MLN4924 or vehicle for 48 h and stimulated for 10 min at 37 °C and 5% CO_2_ with SKO-007(J3) MM cells. A representative experiment is reported (**C**). Histograms represent the results of three independent experiments (**D**). Fluorescence was analyzed using a FACS Canto II flow cytometer (BD Biosciences). Statistical significance was evaluated by ANOVA (*P < 0.05). **E**, **F** Perforin polarization at the IS was analyzed by confocal microscopy in cultivated NK cells, untreated or treated with MLN4924 (150 nM) for 48 h and contacting SKO-007(J3) cells for 15 min. Cells were fixed with paraformaldehyde and after permeabilization were stained for Perforin (in red), F-Actin/Phalloidin (in green) and the nuclei were stained with DAPI (in blue). A representative image of NK/SKO-007(J3) cells conjugates with unpolarized or polarized perforin is shown (**E**). Histograms represent the mean (±SEM) of the % of perforin polarization at IS analyzed on at least 50 conjugates present in randomly acquired fields, from three independent experiments (**P < 0.05) (**F**).

Different other mechanisms could be responsible for the increased degranulation activity of MLN4924-treated NK cells against MM cells. We first explored the possibility that neddylation inhibition could affect the initial steps required for NK cell cytotoxicity following the recognition of the target: the formation of the immunological synapse (IS) and the release of effector molecules. As shown in Supplementary Fig. 11A, B, conjugate formation between CFSE-labeled cultivated NK cells and Cell Tracker Deep Red-loaded SKO-007(J3) cells was not enhanced by MLN4924 treatment at the different time points. We then focused our attention on early signaling events important for cytoskeletal rearrangement and cytotoxic granules release in NK cells. In particular, at the IS, the activity of small GTPases, such as Rac1, RhoA and RhoG, together with other mediators and adaptors, is critical for the efficient degranulation after target cell recognition [41–44]. Indeed, their activity is required for the synaptic F-actin remodeling, and has a strong impact on cytotoxic granules release [41–44]. Of note, Rac1 and RhoA have been identified as substrates of the E3 ubiquitin Cullin-Ring ligases, and their expression and function is increased after neddylation inhibition in different models [44, 50, 51]. To investigate whether the turn-over of these GTPases could be stabilized in our experimental setting, we evaluated the protein expression of Rac1 and RhoA by western blotting in cultivated NK cells exposed to MLN4924. As shown in Supplementary Fig. 12A, neddylation inhibition decreased total active Cullin-NEDD8 conjugates as expected, and this paralleled with a reduced basal degradation (Supplementary Fig. 12B, C) and accumulation of these GTPases (Fig. 6A, B), suggesting a possible higher enzymatic activity in target-activated NK cells, with increased degranulation activity. Accordingly, as shown in Fig. 6C, D, MLN4924 significantly augmented the levels of F-actin in NK cells contacting target cells and increased the efficiency of perforin polarization at the IS in NK cells conjugated to SKO-007(J3) cells, as observed in confocal microscopy (Fig. 6E, F).

### TGFβ-mediated repression of NK cell effector functions is partially abrogated by neddylation inhibition

**Fig. 7:**
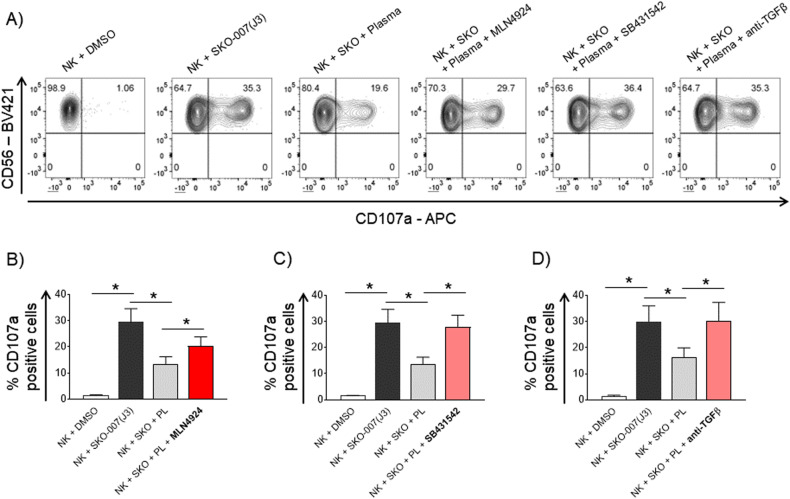
Degranulation activity of NK cells conditioned with bone marrow-derived plasma from MM patients. NK cell degranulation was evaluated using as a source of effector cells PBMCs from healthy donors co-cultured with SKO-007(J3) target cells as described before at E/T ratio 2.5/1 in complete medium at 37 °C and 5% CO_2_ for 2 h. **A** PBMCs were treated for 48 h with MM patient-derived plasma (PL) ± MLN4924 (150 nM), the TGFβ type I receptor inhibitor SB431542 (5 µM), or plasma pre-incubated with an anti-pan/TGFβ mAb 5 µg/ml, prior to the degranulation assay. Cells were stained with Fixable Viability Stain 780, anti-CD14-APC-H7, anti-CD19-APC-H7, anti-CD45-PE-Cy™7, anti-CD3-BV510, anti-CD56-BV421, anti-CD16-PerCP-Cy™5.5 and anti-CD107a-APC. Cell surface expression of CD107a was analyzed on CD14^−^CD19^−^CD45^+^CD3^−^CD56^+^ cells using a FACS Canto II flow cytometer (BD Biosciences) and data were analyzed by FlowJo V10 Cytometric Analysis Software (BD Biosciences). A representative experiment is shown. **B**–**D** Average percentage of CD107a positive cells was calculated based on at least three independent experiments. Statistical significance was evaluated by ANOVA (*P < 0.05).

**Fig. 8:**
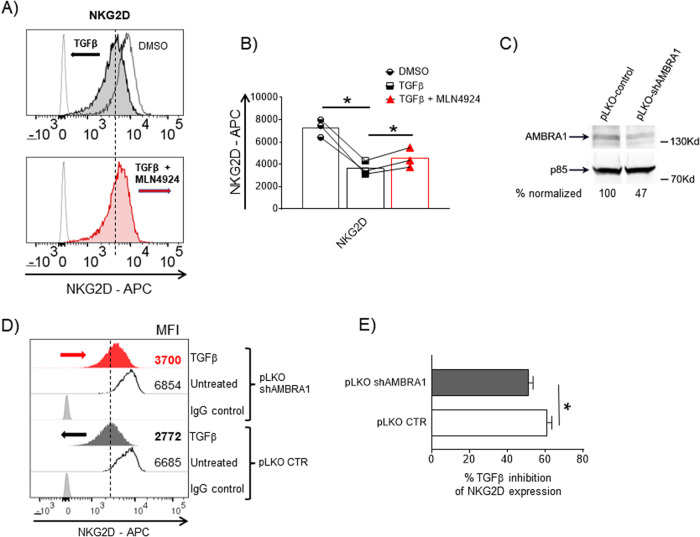
Modulation of AMBRA1 expression and repressive effect of TGFβ on NKG2D expression. **A**, **B** NKG2D expression on NK-L cells was evaluated by flow cytometry after a 48 h treatment with TGFβ 10 ng/ml ± MLN4924 (150 nM). In **A** a representative experiment is shown, with gray filled histograms indicating the expression of the receptor in the presence of TGFβ, and red filled histograms indicating the expression of the receptor in the presence of TGFβ + MLN4924. **B** The MFI values of NKG2D, calculated based on three independent experiments, are shown. **C** Western blot analysis of AMBRA1 in NK-L infected with the lentiviral vector pLKO-shAMBRA1 or control vector. A representative western blot is shown with its densitometric analysis normalized to p85, used as protein loading control. **D**, **E** NKG2D cell surface expression was analyzed by flow cytometry on NK-L-shAMBRA1 cells or control cells, untreated or treated with TGFβ (10 ng/ml) for 48 h. Data are representative of one out of three independent experiments. **E** Histograms represents the mean expression level of NKG2D-APC based on four independent experiments. Statistical significance was evaluated by paired Student’s *t* test (*P < 0.05).

Cancer cells, including MM, can evade immune surveillance and the activity of cytotoxic lymphocytes by secreting a plethora of factors and cytokines. Among these, a major role is played by TGFβ, a cytokine released by cancer cells and other cellular components of the TME. TGFβ is an inhibitor of both adaptive and innate immune responses, and it can support the recruitment and the activity of immunosuppressive cells at the tumor site [13, 52]. Indeed, the activity of NK cells can be strongly affected by TGFβ; in particular, TGFβ can repress IFN-γ production [53] and decrease the surface level of the activating receptors NKG2D and NKp30, reducing the cytotoxic potential of NK cells against tumors [10, 12]. To extend our analysis to the anticancer mechanisms mediated by neddylation inhibition in the tumor microenvironment, we investigated whether it could affect the sensitivity of NK cells to TGFβ and their ability to degranulate against MM cells. We evaluated the activity of BM-derived plasma from MM patients (PL-MM) on the degranulation of NK cells against SKO-007(J3) target cells. As shown in Fig. 7A, B, degranulation of PBMC-derived NK cells pre-treated for 48 h with PL-MM was severely compromised while it was not impaired by plasma from peripheral blood of healthy donors (data not shown). This repression was almost completely abrogated by using a TGFβ type I receptor inhibitor (SB431542) or an anti-pan/TGFβ mAb (Fig. 7C, D), confirming that this cytokine has a major role in the suppression of NK cell functions in the MM BM microenvironment. Noteworthy, in these experiments, the presence of MLN4924 could partially abrogate the effect exerted by PL-MM (Fig. 7B), suggesting a possible action on the TGFβ-mediated repression. To further corroborate this observation, we repeated these experiments by directly using the recombinant cytokine and, in line with the previous experiments, the impairment of NK cell response mediated by TGFβ was partially abrogated by MLN4924 (Supplementary Fig. 13A, B). In this context, we also analyzed the effect of both MLN4924 and TGFβ on NKG2D expression, one of the activating receptors more sensitive to TGFβ [12]. In cultivated NK cells (Supplementary Fig. 14A, B) and in the human NK cell line NK-L (Fig. 8A, B), TGFβ strongly downregulated the expression of this receptor on the cell surface, as already reported [12], and the presence of MLN4924 partially restored NKG2D expression levels. Given the critical role of NKG2D in regulating granule polarization and secretion, these data support the results obtained with the degranulation assays and suggest that the recovery of NKG2D expression could be one of the mechanisms involved. Interestingly, and in line with these data, MLN4924 significantly restored the reduced perforin polarization at the IS in the presence of TGFβ, as observed in confocal microscopy (Supplementary Fig. 14C). Further experiments will be needed to fully characterize the effects of MLN4924 on the regulation of other NK cell activating and inhibitory receptors by TGFβ.

### Neddylation, TGFβ and repression of NK cell effector functions: possible molecular mechanisms involved

Several observations reported that neddylation is involved at various levels in the regulation of TGFβ signaling pathway. Indeed, the E3 NEDD8 ligase c-Cbl can neddylate the TGFβ receptor II (TGFβ-RII), promoting its endocytosis to EEA1-positive early endosomes and preventing its ubiquitination-dependent degradation; this in turn enhances cellular responsiveness to the cytokine [54]. In a different model, AMBRA1, a member of the DDB1 and CUL4-associated factor (DCAF) family of proteins, acts as a substrate receptor for SMAD4. The CRL4^AMBRA1^ ubiquitin ligase, once activated by neddylation, mediates a non-proteolytic polyubiquitylation of Smad4 to enhance its transcriptional functions [55]. Furthermore, NEDD8 targets Smad(s) ubiquitylation regulatory factor 2 (Smurf2) for neddylation promoting its activity but also its ubiquitination and degradation. Smurf2 is a ubiquitin ligase important for the modulation of TGFβ signaling, that interacts with Smad3 and targets Smad1, Smad2 and TGFβ receptor kinases for degradation [56, 57]. In these different models, neddylation inhibition can curtail TGFβ signaling, suggesting that a similar strategy could be effective and ameliorate NK cell responses in the context of the TGFβ-conditioned repressive TME. We investigated the possibility that neddylation inhibition could downregulate cell surface expression of TGFβ-RII, thus reducing the NK cell responsiveness to this cytokine, as already described [54]. However, this was not the case since flow cytometric analysis on gated NK cells from PBMCs exposed to MLN4924 for 48 h showed an unaltered surface expression level of the TGFβ-RII (Supplementary Fig. 15A). These experiments were also repeated at a shorter time point (6 h) with similar results, suggesting that neddylation inhibition does not significantly affect cell surface levels of TGFβ-RII on NK cells (Supplementary Fig. 15B). We also focused our attention to the possible role mediated by AMBRA1 in this modulation, using the NK-L cell line as a model to modulate the expression and the activity of this protein. In this experimental setting, we confirmed the partial restoration of NKG2D levels by MLN4924 in the presence of TGFβ (Fig. 8A, B), and observed that in NK-L cells in which the expression of AMBRA1 was lowered using shRNA interference (Fig. 8C), the repressive effect of TGFβ on NKG2D expression was partially abrogated (Fig. 8D, E), suggesting that the action of CRL4^AMBRA1^ on the transcriptional activity of SMAD4 [55] could represent one of the mechanisms involved in the neddylation mediated regulation of TGFβ signaling in NK cells, and a target of MLN4924.

## Discussion

In this work we investigated how the modulation of neddylation pathway could be a way to boost the anti-MM activity mediated by NK cells. We collected experimental data indicating that inhibition of neddylation enhances NK cell degranulation and killing of MM cells and potentiates Daratumumab and Elotuzumab-mediated ADCC against this tumor. Moreover, neddylation inhibition could partially relieve the repressive effects of TGFβ on NK cell effector activity against MM, suggesting a further mechanism that can unleash the response mediated by these lymphocytes in the context of a strong immunosuppressive TME.

The consequences of neddylation inhibition on the immune responses exerted by NK cells and especially in the TME have not been investigated yet. In this regard, different immunoregulatory outcomes have been described after neddylation inhibition in lymphocytes. Early observations showed a decreased transcription of NF-κB target genes and signaling pathways in TCR-stimulated T cells where NAE was inhibited, while others observed that the activity of neddylated CRLs may in fact negatively affect TCR signaling and IL-2 synthesis [47, 48]. More recently, it has been reported in chronic lymphocytic leukemia (CLL) that patient-derived T cells treated with the NAE inhibitor MLN4924 showed a differential expression of NF‐κB‐regulated genes and downregulated IL-2 expression/signaling during activation [49]. However, the impact of NAE inhibition on overall T-cell activation and proliferation was limited in this model and CD8^+^ T cells maintained their cytotoxicity against both allogeneic and autologous neoplastic lymphoid cells, suggesting that targeting of NAE in the clinic is unlikely to be associated to a severely impaired T-cell functionality. This is a critical point to consider since the consequences of altering the neddylation pathway could result in unpredicted regulatory activities in lymphocytes possibly detrimental for the control of tumor progression. Importantly, NAE inhibition redirected T-cell polarization, preventing the induction of FoxP3^+^ Treg cells and promoting a shift toward IFN-γ-secreting Th1 phenotype [49]. These observations related to the importance of an intact neddylation pathway for Treg cell activity, have been recently corroborated by the evidence that the UBE2M-RBX1 axis and CRL-neddylation is specifically required for intrinsic regulatory processes in these cells. In this regard, small molecule inhibitors of neddylation, such as MLN4924, have been proposed as an effective approach for diseases where Treg cells are over-activated [58]. As Tregs contribute to tumor progression in many cancers, a decreased activity of these cells and a shift toward Th1 cells represents a favorable outcome of NAE inhibition.

In the context of MM biology, our data extend previous pre-clinical observations indicating that inhibition of neddylation could exert different anti-cancer activities. Indeed, overactivation of CRLs has been characterized in tumor growth and progression and the enzymes implicated in the neddylation pathway (e.g., NAE1/UBA3, UBE2M/UBE2F, NEDD8 E3 ligases) are often overexpressed in different human cancers, including MM [59]. Here, neddylation represents a druggable pathway, the modulation of which has been described to overcome stroma and osteoclast protection of MM cells and to repress MM proliferation in xenograft murine models [59]. Moreover, in MM cells, neddylation restrains the expression of NK cell activating ligands and its inhibition sensitizes these cells to NK cell recognition and killing [40]. Our data add a novel piece of information on the impact of neddylation on NK cell anti-cancer activity against MM and on the interplay with the repressive effects mediated by TGFβ on their activity in the context of MM TME.

Inhibition of neddylation potentiated natural (Figs. 1 and 2, and Supplementary Figs. 2 and 4) and antibody-mediated degranulation of NK cells against MM cells (Figs. 3 and 4, and Supplementary Figs. 6–8), where Daratumumab and Elotuzumab are fully human and humanized monoclonal antibodies targeting CD38 and SLAMF7/CS1 respectively, approved for the treatment of MM [46, 60]. These data were confirmed using as effectors NK cells from both PBMCs and CD138^−^ BMMCs, after depletion of autologous MM cancer cells. This suggests the absence of possible or unpredicted negative effects of neddylation inhibition on ADCC mediated by NK cells against MM also in the clinic. Inhibition of neddylation did not significantly alter the induction of cytokines such as TNF-α or IFN-γ, neither the overall expression of cytotoxic molecules in NK cells contacting MM target cells (Fig. 5); moreover, only slight differences were detected in the expression of activating and inhibitory receptors mostly involved in NK cell-mediated immunosurveillance (Supplementary Figs. 9 and 10). Interestingly, although the inhibition of neddylation did not affect conjugate formation between cultivated NK cells and SKO-007(J3) MM cells (Supplementary Fig. 11), it significantly upregulated the expression levels of Rac1 and RhoA GTPases, two mediators of early signaling events important for cytoskeletal rearrangements and cytotoxic granule release after target recognition [41–44] (Fig. 6A, B). Of note, and in line with our data, both Rac1 and RhoA have been characterized as substrates of Culling-Ring ligases in different models [44, 50, 51, 61] and targets of MLN4924. Thus, the increased levels of these GTPases can augment actin dynamics and degranulation of activated NK cells. As a confirm, treatment with MLN4924 increased the levels of F-actin in NK cells contacting target cells (Fig. 6C, D), a mechanism involved in regulating the cytoskeletal dynamics and degranulation. Moreover, MLN4924 increased the % of perforin polarization at the IS (Fig. 6E, F). In this regard, our data seem to be consistent with a recent work from other laboratories exploring GTPases-associated pathways affected in Hemophagocytic Lymphohistiocytosis (HLH) with deficiency of RhoG, where lymphocyte exocytosis is abrogated without impairment of stable conjugates formation, activating receptors signaling and cytokine production. The authors identified MLN4924 as a small-molecule inhibitor able to upregulate and rescue the impaired Rac1 activation in RhoG-deficient NK (and T) cells, and partially reverse their compromised F-actin remodeling and degranulation [44]. Further experiments will be needed to better investigate and confirm this mechanism in our experimental system.

MM conditioned TME represents a major barrier for an efficient control of tumor progression by cytotoxic lymphocytes, including NK cells. We focused our attention on the massive presence of TGFβ, a cytokine secreted by cancer cells and whose expression is induced by these in the neighboring cells. In MM, TGFβ is abundantly released and activated at the level of the destructive bone lesions and plays a major role in the suppression of bone formation [7]. Moreover, TGFβ is a powerful inhibitor of both the adaptive and innate immune responses, further supporting tumor progression [13, 52]. The finding that treatment with MLN4924 could partially revert the repressive effects of TGFβ on NK cells effector activity against MM (Fig. 7 and Supplementary Fig. 13), in addition to the aforementioned direct boost of their degranulation activity, further extends the therapeutic potential of neddylation inhibition in this context. In addition, MLN4924 restored the reduced level of perforin polarization at the IS in the presence of TGFβ, as observed in confocal microscopy (Supplementary Fig. 14C), and the expression level of NKG2D, a critical NK cell activating receptor for MM recognition and killing [17, 23], the expression of which is severely inhibited by TGFβ [10, 12] (Fig. 8 and Supplementary Fig. 14).

Neddylation can affect different steps of the TGFβ signaling pathway. The E3 NEDD8 ligase c-Cbl can neddylate TGFβ-RII, preventing its ubiquitination-dependent degradation, and this can enhance cellular responsiveness to the cytokine [54]. Moreover, AMBRA1, a member of the DCAF family of proteins is a substrate receptor for Smad4, mediating as part of a neddylated/activated CRL4^AMBRA1^ complex a non-proteolytic polyubiquitylation of Smad4 to enhance its transcriptional functions [55]. In a different model, NEDD8 has been described to target Smurf2, a ubiquitin ligase important for the modulation of TGFβ signaling [56, 57], promoting its function, but also accelerating its degradation. Thus, neddylation can influence TGFβ signaling and, in this way, promote a strong suppressive TME with a consequent repression of the immune response against tumors. Our data indicate that MLN4924 does not significantly change cell surface expression of TGFβRII on NK cells (from PBMCs) (Supplementary Fig. 15), nor the levels of Smurf2 in cultivated NK cells (data not shown), suggesting that these two pathways are not involved in the modulation of TGFβ activity played by MLN4924, in our experimental system. On the contrary, we observed that in NK-L cells, in which the expression of AMBRA1 was lowered by shRNA interference, the repressive effect of TGFβ on NKG2D expression was partially abrogated (Fig. 8C–E), suggesting that the action mediated by CRL4^AMBRA1^ on the transcriptional activity of SMAD4 [55] could represent a mechanism involved in the neddylation-mediated regulation of TGFβ signaling in NK cells and a target of MLN4924 in this setting. Further experiments will be required to better address this possibility. Of note, and given the relevance of TGFβ in regulating Treg cell biology in cancer [62], AMBRA1 has been shown to increase the Treg response in different pathological conditions, including tumor immunosurveillance and progression [63].

**Fig. 9:**
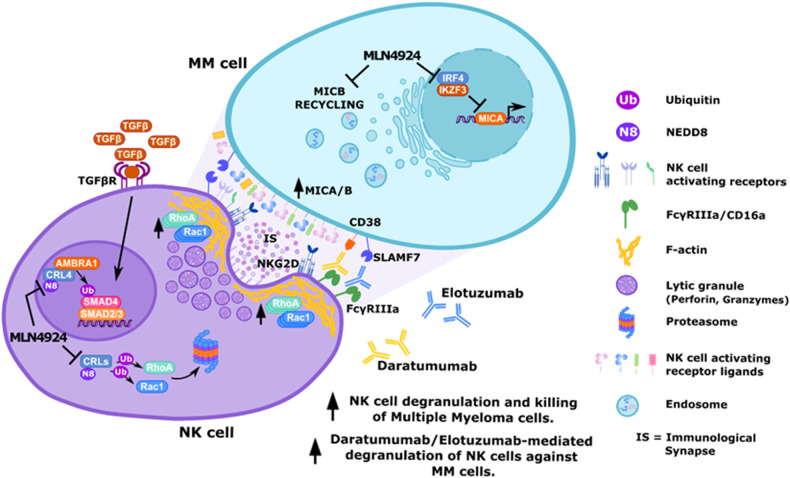
Model: inhibition of neddylation and stimulation of NK cell activity against MM. Inhibition of neddylation by MLN4924 potentiates NK cell-mediated degranulation and cytotoxic activity against MM. Correlation with accumulation of RhoA/Rac1 GTPases and increased levels of F-actin and perforin polarization in NK cells contacting target cells. Inhibition of neddylation can restrain TGFβ-mediated negative regulation of NK cell cytotoxic function and NKG2D expression, which contributes to the suppressive TME. In addition, as already described, inhibition of neddylation can increase NKG2D ligand MICA and MICB expression on the tumor side, sensitizing these cells to NK cell recognition and killing [40].

Altogether our results extend the knowledge about the immunoregulatory activities mediated by neddylation in the context of MM (Fig. 9), extending our previous observations on the upregulation of the NKG2D ligand MICA and MICB in MM cells by inhibitors of neddylation, sensitizing these cells to NK cell recognition and killing [40]. These results further encourage future studies for the identification and use of novel inhibitors of this pathway, even more specialized or redirected to selected classes of CRLs or non-cullin targets, for more effective immunotherapeutic anti-cancer strategies.

## Materials and methods

### Cell lines and clinical samples

The NK cell lines NK-L and NK92 were cultured at 37 °C and 5% CO_2_ in RPMI 1640, 10% FBS (GIBCO, Life Technologies, Gaithersburg, MD), 2 mM L-glutamine, 100 U/ml penicillin and 100 U/ml streptomycin (complete medium) supplemented with 200 U/ml of IL-2 (PeproTech, London, UK). The human myeloma cell lines SKO-007(J3), RPMI-8226 and ARK have been already described [64] and were kindly provided by Prof. P. Trivedi (University of Rome, Sapienza, Italy). Cells were cultured at 37 °C and 5% CO_2_ in complete medium for no longer than 4 weeks and tested for mycoplasma monthly [Mycoplasma PCR Detection Kit, Applied Biological Materials Inc. (ABM) - Richmond, BC, Canada]. MM cell lines were authenticated by IRCCS Azienda Ospedaliera Universitaria San Martino-IST, S.S. Banca Biologica e Cell factory (Genova, IT). The human 293 T embryonic kidney cell line was purchased from ATCC and was maintained in Dulbecco’s modified Eagle’s supplemented with 10% FBS, 2 mM L-glutamine, 100 U/ml penicillin and 100 U/ml streptomycin. Bone marrow samples from MM patients were managed at the Division of Hematology, Department of Cellular Biotechnologies and Hematology, University of Rome, Sapienza, Italy (Table 1). Informed consent in accordance with the Declaration of Helsinki was obtained from all patients, and approval was obtained from the Ethics Committee of the Sapienza University of Rome (Rif. 5191). Bone marrow mononuclear cells (BMMCs) were isolated from bone marrow aspirates by Ficoll–Hypaque density gradient centrifugation (Lympholyte Cedarlane, Burlington, Ontario, Canada). In some experiments, BMMCs were depleted of myeloma cells, by negative selection, using anti-CD138 magnetic beads (Miltenyi Biotec. S.R.L. Bologna, IT) and used as effector cells (referred as the CD138^−^ fraction). On the other hand, purified myeloma cells (more than 90% of them expressing CD138 and CD38) were used as targets in autologous experiments. Peripheral blood mononuclear cells (PBMCs) from healthy donors were isolated from blood samples by Ficoll–Hypaque density gradient centrifugation and used as effector cells. Cultivated human NK cells were obtained after 10-day co-cultures of PBMCs with irradiated Epstein-Barr virus positive (EBV^+^) RPMI 8866 lymphoblastoid cell line as already described in [65]. On day 10, the cell population was routinely >90% CD56^+^CD16^+^CD3^−^, as assessed by immunofluorescence and flow cytometry analysis.

**Table 1:** Clinical parameters of MM patients.

Patient no.	Sex/Age	Clinical stage	Monoclonal Ig	% PCs in BM
1	F/83	ONSET	IgA-λ	19
2	M/69	RELAPSE	IgG-λ	2
3	M/78	ONSET	IgG-λ	15
4	F/73a	RELAPSE	IgG-k	31
5	F/75	RELAPSE	IgG-k	40
6	F/82	ONSET	IgG-k	62
7	M/52	ONSET	IgA-λ	56
8	M/68	ONSET	IgG-k	17
9	F/66	RELAPSE	Micro-k	82
10	M/73	ONSET	IgG-k	6
11	F/81	SMOLDERING	IgG-λ	23
12	M/73	RELAPSE	IgA-k	40
13	F/67	RELAPSE	IgG-k	10
14	M/66	ONSET	IgG-k	45
15	F/85	ONSET	IgG-k	13
16	M/72	ONSET	IgG-λ	37
17	M/66	RELAPSE	IgG-k	7

Patients were classified according to the status of disease. The percentage of plasma cells in the BM and monoclonal Ig is indicated.

### Reagents and antibodies

The NAE inhibitor MLN4924/Pevonedistat was purchased from Selleckchem.Com (Houston, Texas, USA). The TGFβ type I receptor inhibitor SB431542 and Cycloheximide (CHX) were purchased from Sigma-Aldrich (St. Louis, MO, U.S.A.). The final concentration of DMSO in all experiments was <0.1%. Daratumumab and Elotuzumab mAbs were provided by the “Policlinico Umberto I” hematology center pharmacy.

The following monoclonal antibodies (mAbs) were used for immunostaining: anti-CD14-APC-H7 (MφP9), anti-CD19-APC-H7 (HIB19), anti-CD45-PE-Cy™7 (HI30), anti-CD3-BV510 (HIT3a and UCHT1), anti-CD56-BV421/PE (NCAM16.2), anti-CD16-PE-Cy™7 (3G8), anti-CD107a-APC (H4A3), anti-CD138-FITC/PE (MI15), anti-CD38-APC (HIT2), anti-IFN-γ-PE (4S.B3), anti-Perforin-Alexa Fluor® 488 (δG9), anti-Granzyme B-PE (GB11), anti-NKG2D-APC (1D11), anti-DNAM1-FITC (DX11), anti-NKp30-Alexa Fluor® 647/BB700 (p30-15), anti-TIM-3-BB515 (7D3), anti-PD-1-BV421 (EH12.1) were purchased from BD Biosciences (Franklin Lakes, New Jersey, U.S.A.). Anti-NKp46-PE (9E2) and anti-SLAMF7-APC (162.1) were purchased from BioLegend (San Diego, California, U.S.A.) . Anti-TNFα-FITC (cA2), REA Control Antibody-APC (REA293), anti-TGFβ RII-APC (REA903) were purchased from Miltenyi Biotec (Bergisch Gladbach, North Rhine-Westphalia, Germany). Anti-TGFβ_1/2/3_ (1D11) monoclonal antibody was used as blocking antibody [R&D System (Minneapolis, MN, USA)].

### Flow cytometry: degranulation assays

Degranulation assays were performed using PBMCs from healthy donors or BMMCs from bone marrow aspirates depleted of MM plasma cells (from now referred as CD138^−^ cells) as source of NK effector cells. PBMCs were cultured (in 24 or 12-well tissue culture plates) at 10^6^ cells/ml in complete medium supplemented with 100 U/ml of IL-2 and treated with MLN4924 (150 nM) or vehicle (DMSO) for 72 h. Cells were then (washed in complete medium and) co-cultured with untreated SKO-007(J3), RPMI 8226 or ARK target cells [Effector:Target (E:T) ratio of 2.5:1] for 2 h at 37 °C and 5% CO_2_. Thereafter, cells were washed with phosphate-buffered saline (PBS) 2% FBS and incubated with anti-CD14-APC-H7, anti-CD19-APC-H7, anti-CD3-BV510 (from now on mentioned as lin^−^), anti-CD45-PE-Cy™7, anti-CD56-BV421, anti-CD16-PerCP-Cy™5.5 and the degranulation marker anti-CD107a-APC for 45 min at 4 °C. In some experiments, RPMI 8226 and ARK target cells were pre-treated with anti-CD38/Daratumumab 1 µg/10^6^ cells for 15 min at room temperature and then washed twice in complete medium. In other experiments anti-SLAMF7/Elotuzumab has been added to the co-culture at the final concentration of 1 µg/10^6^ cells, during the incubation of 2 h at 37 °C and 5% CO_2_. CD107a cell surface expression was evaluated on the NK cell population, gated as CD56^+^CD16^+/−^, using a FACS Canto II flow cytometer (BD Biosciences). CD138^−^ cells were cultured in complete medium supplemented with IL-2 (200 U/ml) at 3 × 10^6^ cells/ml and treated with MLN4924 or vehicle for 48 h. For CD107a cell surface expression, cells were co-cultured with SKO-007(J3), RPMI 8226, ARK MM cells or autologous patient-derived enriched plasma cells, untreated or, in some cases, pre-treated with Daratumumab or treated with Elotuzumab (as described above) (E:T ratio of 2.5:1) for 2 h at 37 °C and 5% CO_2_. Then cells were washed with PBS 2% FBS and incubated with lin^-^, anti-CD45-PE-Cy™7, anti-CD56-BV421, anti-CD16-PerCP-Cy™5.5 to gate NK cells, anti-CD138-PE/FITC to exclude PCs and the degranulation marker anti-CD107a-APC for 45 min at 4 °C. Samples were acquired using FACS Canto II flow cytometer. In these experiments, autologous MM PCs were cultured (after purification) in complete medium supplemented with the addiction of IL-3 (20 ng/ml) and IL-6 (2 ng/ml) at 1 × 10^6^ cells/ml.

### Flow cytometry: analysis of effector molecules and receptor expression

The expression levels of the cytokines IFN-γ and TNF-α and the cytotoxic proteins Perforin and Granzyme B, were analyzed in PBMCs treated with the drug or vehicle, and co-cultured with SKO-007(J3) target cells (E:T ratio 2.5:1) for 6 h at 37 °C and 5% CO_2_. After one-hour Brefeldin A (5 µg/ml) and Monensin (25 µM) (Sigma-Aldrich) were added. Thereafter, cells were washed with PBS 2% FBS and incubated with lin^-^, anti-CD45-PE-Cy™7, anti-CD56-BV421, anti-CD16-PerCP-Cy™5.5. After extracellular staining, cells were fixed and permeabilized using BD Cytofix/Cytoperm™ Fixation/Permeabilization Kit (BD Biosciences) and stained with anti-IFN-γ-PE, Anti-TNFα-FITC, anti-Perforin-Alexa Fluor® 488, anti-Granzyme B-PE. Fluorescence was analyzed, on gated NK cells, using a FACS Canto II flow cytometer (BD Biosciences).

The expression of different NK cell activating, and inhibitory receptors was analyzed, on gated NK cells from PBMCs (lin^-^, anti-CD45-PE-Cy™7, anti-CD56-BV421/PE^+^, anti-CD16-PerCP-Cy™5.5^+/^) and on cultivated NK cells (CD56^+^CD16^+^CD3^−^) from healthy donors, treated with the drug. Cultivated NK cells were cultured at 10^6^ cells/ml in complete medium and stimulated with MLN4924 (150 nM) or vehicle for 48 h. Receptor immunofluorescent staining was performed using the following directly conjugated monoclonal antibodies/mAbs: anti-NKG2D-APC, anti-DNAM1-FITC, anti-NKp30-Alexa Fluor® 647/BB700, anti-NKp46-PE, anti-TIM-3-BB515, anti-PD-1-BV421 and analyzed through a FACS Canto II flow cytometer (BD Biosciences). Non-specific fluorescence was assessed by using an isotype-matched control Ig. In all experiments, cells were stained with BD Horizon™ Fixable Viability Stain 780 (BD Biosciences) to assess cell viability and exclude dead cells. To calculate the averages of the different mean fluorescence intensities (MFI) of each sample/treatment, the corresponding MFI of the control isotype was always subtracted from the MFI of the specific mAb (for that treatment). Data were analyzed by FlowJo V10 Cytometric Analysis Software (BD Biosciences, San Jose, California, USA).

### Flow cytometry: cytotoxicity assay, conjugate formation assay, F-actin staining and activity of patient-derived plasma on NK cells

To test NK cell-mediated cytotoxicity, cultivated NK cells from healthy donors (untreated or treated with MLN4924) were incubated with SKO-007(J3), previously labeled with 5(6)-Carboxyfluorescein diacetate N-succinimidyl ester (CFSE) (2,5 µM) (Sigma-Aldrich), at different Effector:Target (E:T) ratios, for 4 h at 37 °C. Cells were then washed with PBS 1% BSA and stained with 7-Aminoactinomycin D (7-AAD) (Sigma-Aldrich) at the final concentration of 5 µg/ml for 20 min at 4 °C. Specific lysis of target cells has been analyzed using a FACS Canto II flow cytometer (BD Biosciences).

To analyze conjugate formation, CFSE-labeled NK cells were allowed to form conjugates with DEEP RED-labeled (CellTracker™ Deep Red dye™ - Invitrogen - Waltham, Massachusetts, USA) SKO-007(J3) (E:T ratio 2:1) for 15 and 30 min at 37 °C. Cells were then fixed with 1% paraformaldehyde for 20 min on ice, washed with cold PBS and sample were acquired at FACS Canto II flow cytometer. Double-positive cells were intended as conjugates and the percentage of conjugates was determined as NK cells bound to targets out of the total NK cells.

To check the cellular levels of F-actin, cultivated NK cells, treated with MLN4924 or vehicle for 48 h, were activated/stimulated with SKO-007(J3) MM cells (E/T 1/1), previously immobilized on a poly-L-Lysine-treated MW48 (Sigma-Aldrich), for 10 min at 37 °C and 5% CO_2_. Effector cells were then retrieved, fixed and permeabilized using BD Cytofix/Cytoperm™ Fixation/Permeabilization Kit (BD Biosciences) and stained with Phalloidin-Alexa Fluor® 488 (Invitrogen, Waltham, Massachusetts, USA) for 30 min at 4 °C. F-actin levels have been analyzed using a FACS Canto II flow cytometer. To evaluate the inhibitory effect of TGFβ from MM patient-derived plasma on NK cell degranulation, PBMCs (from healthy donors) were exposed to bone marrow patient-derived plasma in the presence of MLN4924 or vehicle. Plasma was collected after centrifugation of the whole (heparinized) blood samples at 1400 rpm for 10 min and stored a −80 °C. Before use, a further clarification of the plasma was achieved through centrifugation at 16.000 g for 10 min. To assess the contribution of TGFβ on the impairment of NK cell degranulation mediated by patient-derived plasma, PBMCs were treated with plasma and the TGFβ type I receptor inhibitor SB431542 (5 µM) or with plasma pre-incubated with an anti-pan/TGFβ mAb (5 µg/ml) for 20 min at 4 °C and with constant agitation. In some experiments, plasma from the peripheral blood of healthy donors was used as control. To investigate the effect of recombinant TGFβ and MLN4924 treatment on NK cell degranulation, PBMCs were cultivated with recombinant TGFβ (10 ng/ml) (PeproTech, London, UK) in the presence of MLN4924 (150 nM) or vehicle for 72 h. In all these assays NK cell degranulation was evaluated by co-culturing PBMCs with SKO-007(J3) (E/T 2.5/1) for 2 h as described above.

### Confocal microscopy

Cultivated NK cells, stimulated 48 h with MLN4924 150 nM or left untreated, were co-cultured with SKO-007(J3) (E:T ratio 2:1) at 37 °C 15 min in multichambered slides (Falcon) pre-coated with poly-L-lysine (Sigma-Aldrich) and then let adhere by centrifugation at 100 g. Cells were then fixed with 4% paraformaldehyde, treated with Glycine 0.1 M for 20 min to quench PFA and permeabilized with 0.1% Triton-X-100 for 5 min. Cells were stained with anti-Perforin mAb (δG9) (Ancell Corporation - Bayport, Minnesota, United States) for 1 h followed by Alexa Fluor 594-conjugated goat anti-mouse IgG2b (A21145 - Invitrogen, Life Technologies) for 1 h and with Alexa Fluor 488-cojugated Phalloidin (A12379 - Invitrogen, Life Technologies) for 30 min all diluted in blocking buffer (PBS 0.01% Triton-X-100, 1% FBS). After extensive washing coverslips were mounted using SlowFade Gold Antifade Mountant with DAPI (S36938 – Thermo Fisher Scientific). High-resolution images (1024 × 1024 pixel, 4,10 μs/pixel, zoom 3x) were acquired at room temperature using Zeiss LSM 980 confocal microscope with a Plan-Apochromat 63x/1.40 NA oil immersion objective (all from Zeiss – Jena Germany). Images were processed with ZEISS ZEN 3.7 Software. Data analysis was performed with ZEISS ZEN 3.7 Software.

### Plasmids, virus production and in vitro transduction

For knocking down AMBRA1, NAE1(APPBP1) and UBA3, we used the following lentiviral vectors/sequences: pLKO.1-sh-AMBRA1 (TRCN0000168355), (TRCN0000168652), pLKO.1-sh-NAE1 (TRCN0000007240), (TRCN0000007242), pLKO.1-sh-UBA3 (TRCN0000007254), (TRCN0000007255), and the control vector pLKO/puro non-targeting shRNA (MISSION™ Sigma-Aldrich). For lentivirus production, lentiviral vectors were co-transfected together with the packaging vectors pVSVG and psPAX2 into 293 T cells using Lipofectamine 2000 (Invitrogen - Waltham, Massachusetts, USA). After transfection, cells were placed in fresh medium. After a further 48-hour culture, virus-containing supernatants were harvested, filtered and used immediately for infections. Spin-infections were performed on 0.3 × 10^6^ NK-L or NK92 cells/ml of viral supernatant containing IL-2 (500 U/ml) and Polybrene (8 μg/ml) (Hexadimethrine bromide - Sigma-Aldrich) at 1800 rpm for 45 min. Two infection cycles were performed. After 48 h, infected cells were grown in selection media containing puromycin at 1 μg/ml.

### mRNA detection and quantitative real-time polymerase chain reaction (qRT-PCR)

Total RNA was extracted using Total RNA Mini kit (Geneaid Biotech, New Taipei City, Taiwan), according to manufacturer’s instructions. The concentration and quality of the extracted total RNA was determined by measuring light absorbance at 260 nm (A260) and the ratio of A260/A280. Reverse transcription was carried out in a 25 µl reaction volume with 2 µg of total RNA according to the manufacturer’s protocol for M-MLV reverse transcriptase (Promega, Madison, Wisconsin, USA). cDNAs were amplified (TaqMan assays) in triplicate with primers for NAE1 (Hs01000370_m1), UBA3 (Hs01091470_m1) and GAPDH (Hs02758991_g1) conjugated to the fluorochrome FAM (Applied Biosystems, Foster City, California, USA). The expression level was measured using the comparative Ct (threshold cycle) method. ΔCt was obtained by subtracting the Ct value of the gene of interest from the selected housekeeping gene (GAPDH) Ct value. In the present study, we used the ΔCt of the control sample as calibrator. The fold change was calculated according to the formula 2^−ΔΔCt^, where ΔΔCt was the difference between ΔCt of the sample and the ΔCt of the calibrator (according to the formula, the value of the calibrator in each run is 1). All PCR reactions were performed using an ABI Prism 7900 Sequence Detection system (Applied Biosystems).

### Western-Blot analysis

For Western-Blot analysis, cultivated NK cells or NK-L cells, treated with MLN4924 or vehicle for 48 h, were pelleted, washed once with cold PBS, resuspended in lysis buffer [1 mM EDTA, 50 mM Tris-HCl pH 7.6, 150 mM NaCl, 0,2% Triton X-100, 0,3% Nonidet P-40 (NP-40), 50 mM NaF, 1 mM Na_3_VO_4_, 1 mM PMSF, Protease Inhibitor Cocktail 1X (Sigma Aldrich, St. Louis, Missouri, USA), Phosphatase Inhibitor Cocktail 3 1X (Sigma Aldrich)] and then incubated 20 min on ice. The lysates were centrifuged at 16.000 g for 20 min at 4 °C and the supernatants were collected as whole cell extract. Protein concentration was determined through the BCA method (Pierce - ThermoFisher Scientific, Milan, IT). 15–30 μg of cell extracts were run on 7 or 12% denaturing SDS-polyacrylamide gels. Proteins were then electroblotted onto Amersham™ Protran™ nitrocellulose membranes (GE Healthcare Life Science, Chicago, Illinois, USA), stained with Ponceau to verify that similar amounts of proteins had been loaded in each lane, and blocked with 5% BSA in TBST buffer. Immunoreactive bands were visualized on the nitrocellulose membranes, using horseradish-peroxidase-linked/coupled donkey anti-rabbit (NA934V) or sheep anti-mouse (NA931V) IgG (Amersham, GE Healthcare Life Science) and the ECL substrate WESTAR ηC ULTRA 2.0 (Cyanagen, Bologna, Italy), following the manufacturer’s instructions. The following antibodies were used: anti-Rac1 (23A8) and anti-p85 (ABS233) were purchased from Millipore (Burlington, Massachusetts, USA). Antibodies anti-Rho A (26C4) and AMBRA1 (G-6) were purchased from Santa Cruz Biotechnology (Dallas, Texas, USA). Antibody anti-NEDD8 (19E3) was purchased from Cell Signaling (Danvers, Massachusetts, USA). Antibody anti-β-Actin (AC-15) was purchased from Sigma-Aldrich. The iBright Analysis Software (Thermo Fisher Scientific - Waltham, Massachusetts, U.S.) was used for densitometric analysis of the gels acquired using iBright™ CL1500 Imaging System (Thermo Fisher Scientific). Target protein levels were referred to β-Actin or p85, chosen to normalize protein expression.

### Statistical analysis

Error bars represent SEM. Data have been evaluated by paired Student’s *t* test or ANOVA using GraphPad Prism 8, and a *P* < 0.05 was considered statistically significant.

## Supplementary information

Suppl. Figures

Suppl. Fig. Legends

Uncropped WBs

## Data Availability

All data generated or analyzed during this study are included in this published article and its supplementary information files.
