# Supplementary figures and images for: NEDD8-activating enzyme inhibition potentiates the anti-myeloma activity of natural killer cells
Source: Cell Death Dis. 2023 Jul 17;14(7):438. doi: 10.1038/s41419-023-05949-z (PMC10352239; doi:10.1038/s41419-023-05949-z)

## Gating Strategy - PBMCs

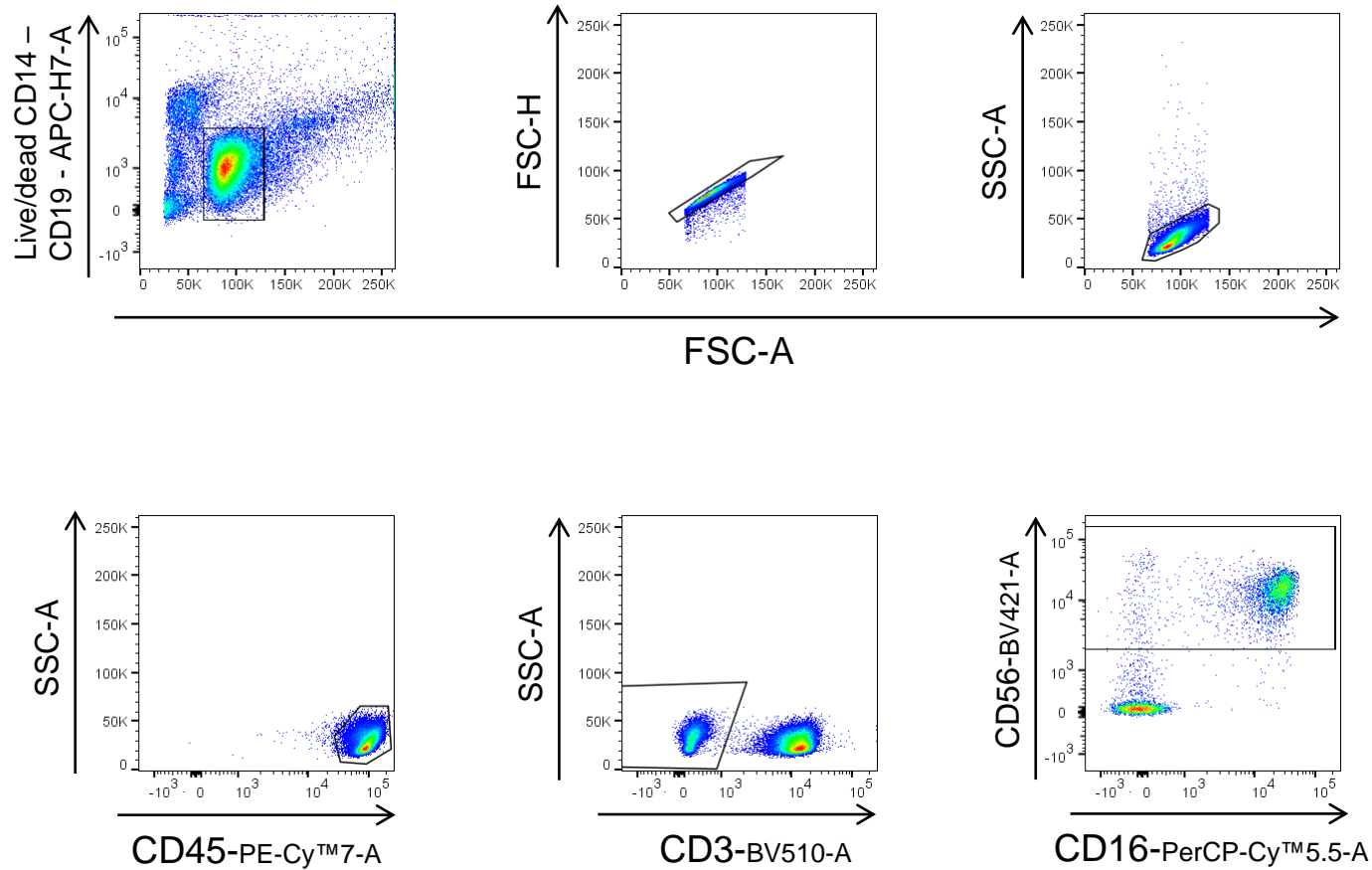

A)

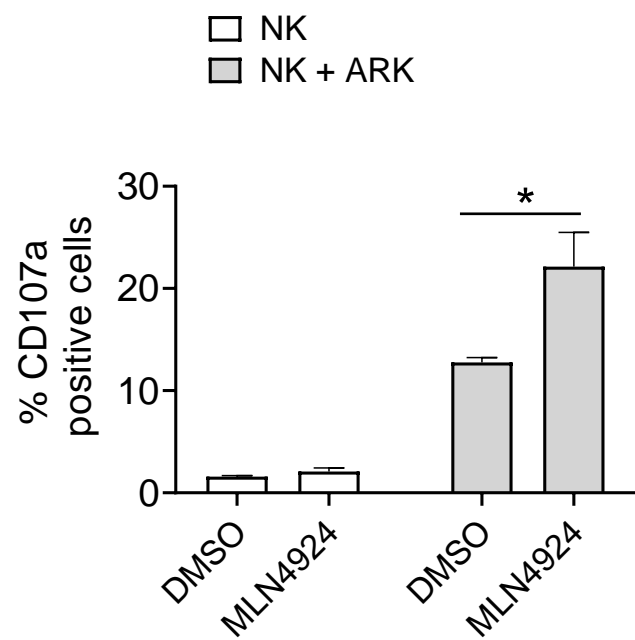

B)

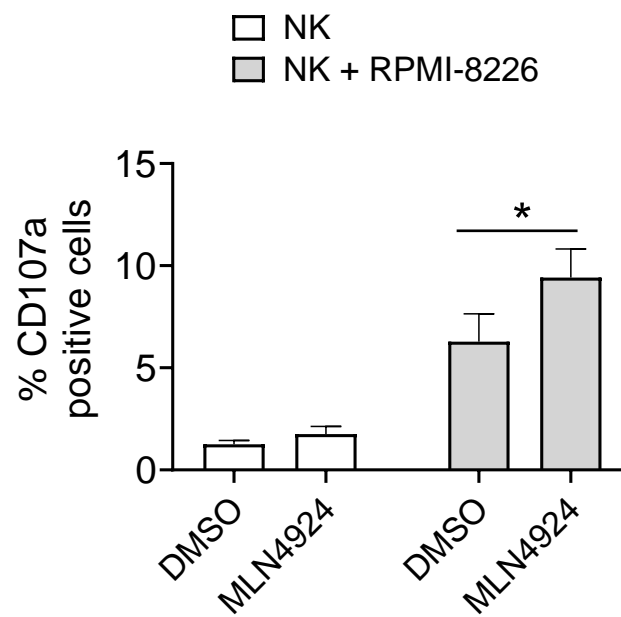

## Gating Strategy – NK / BMMCs

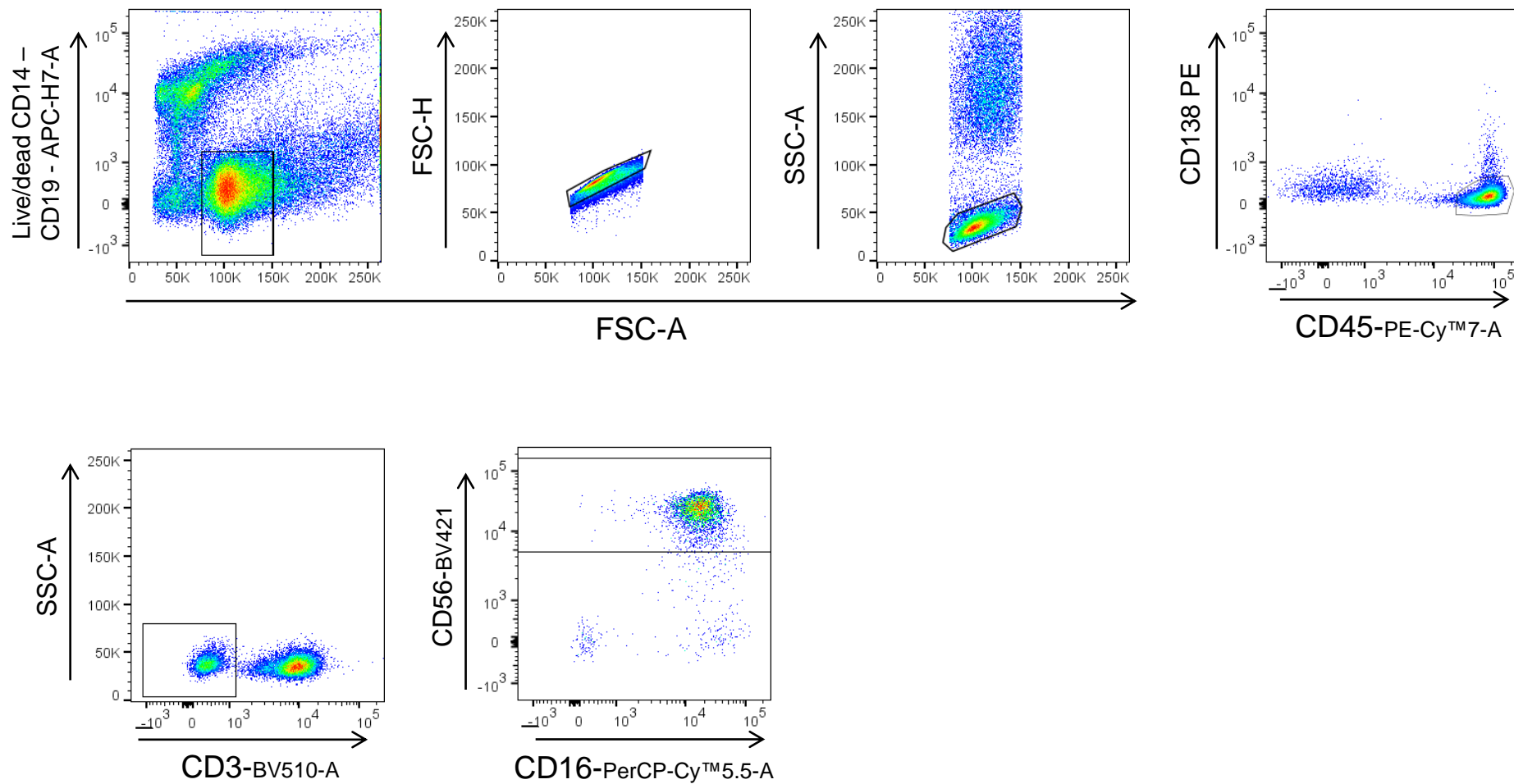

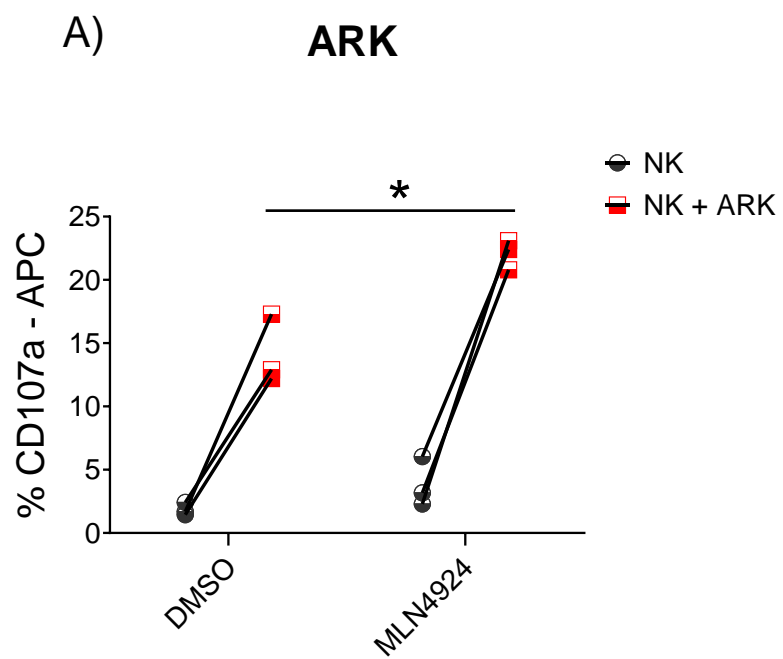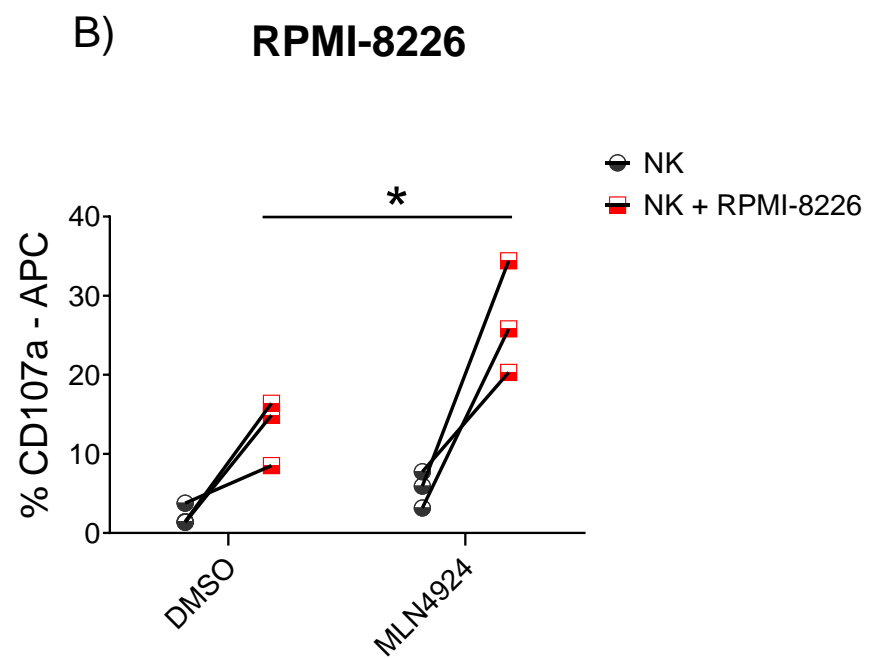

A)

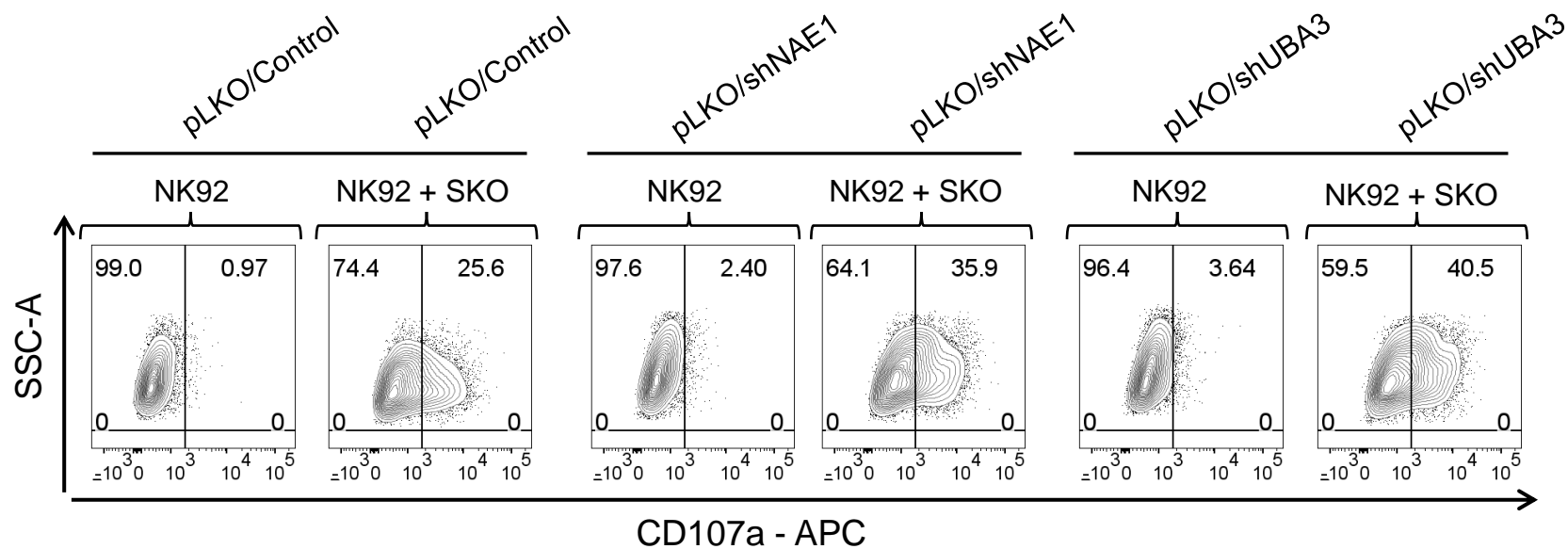

B)

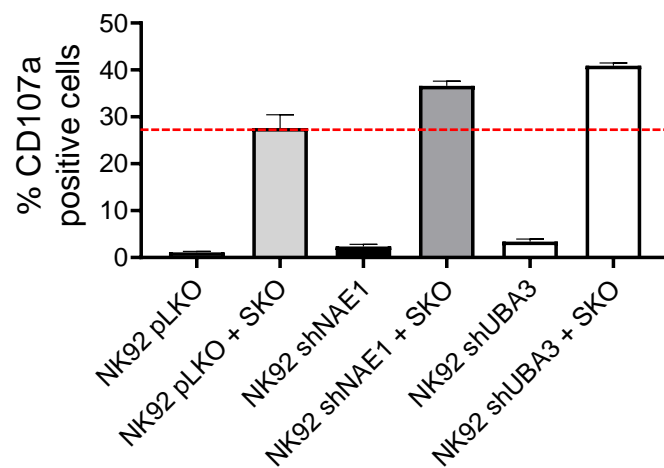

C)

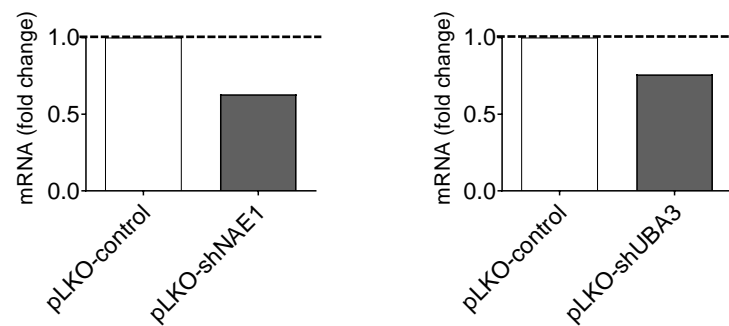

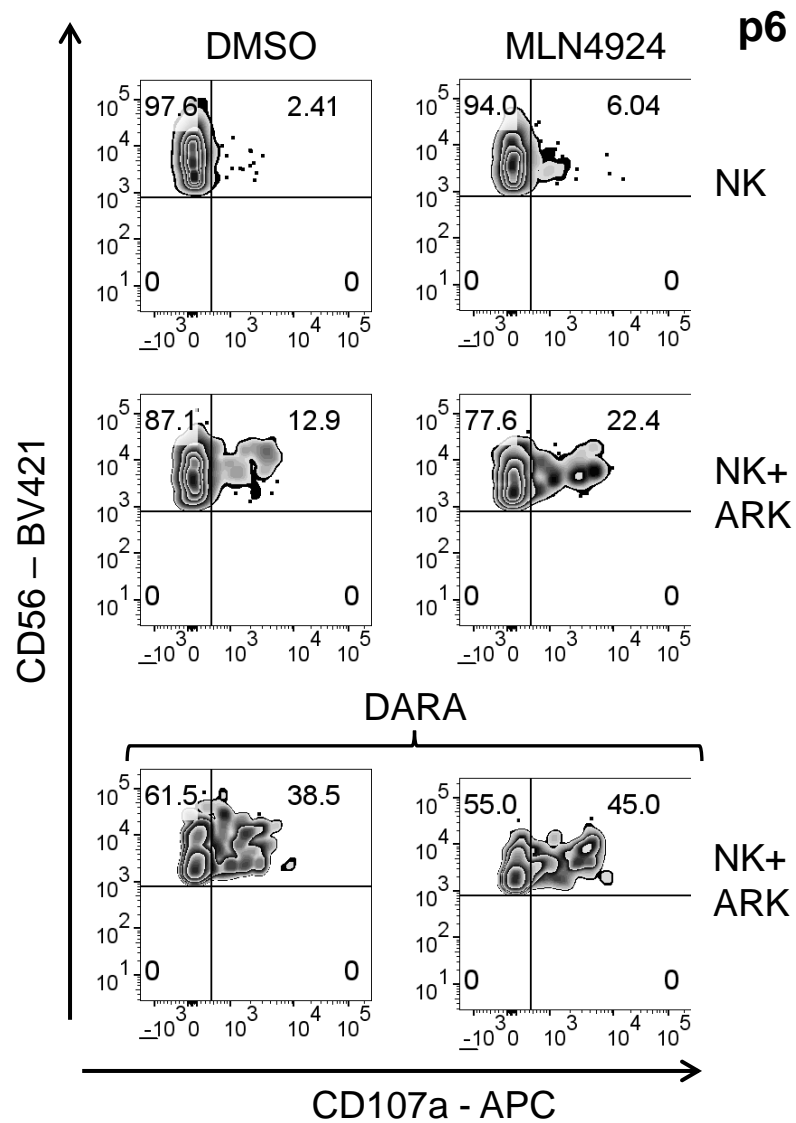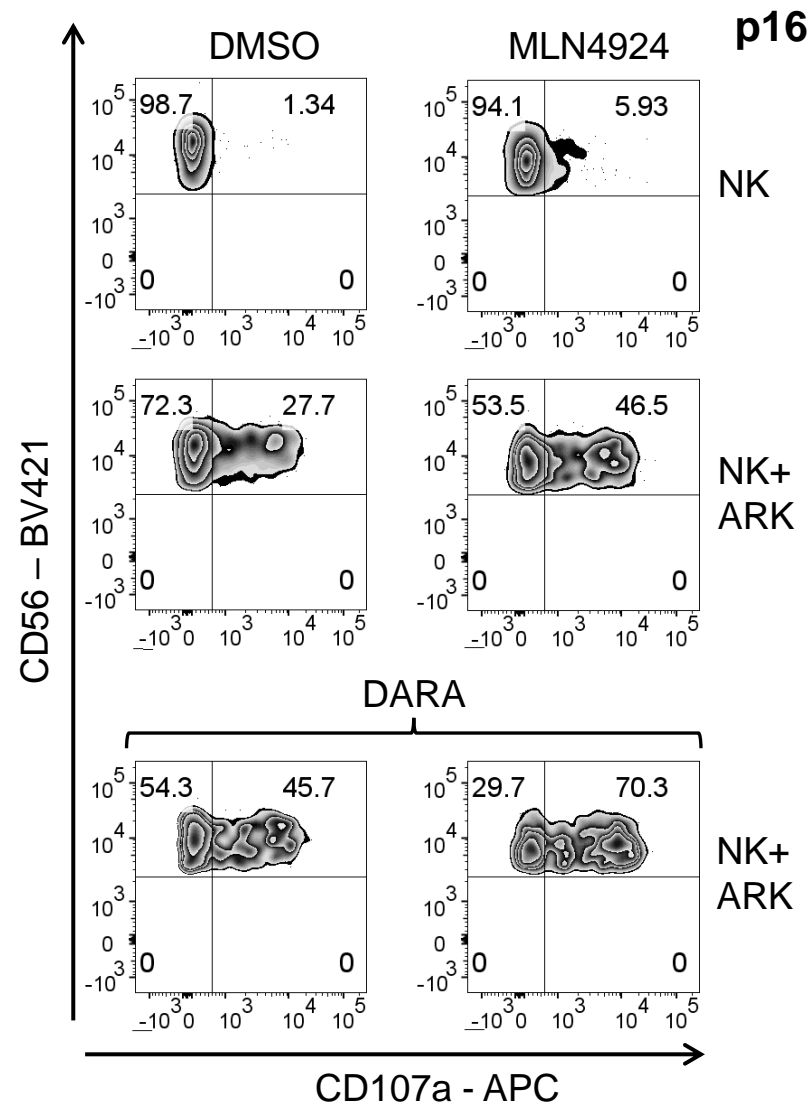

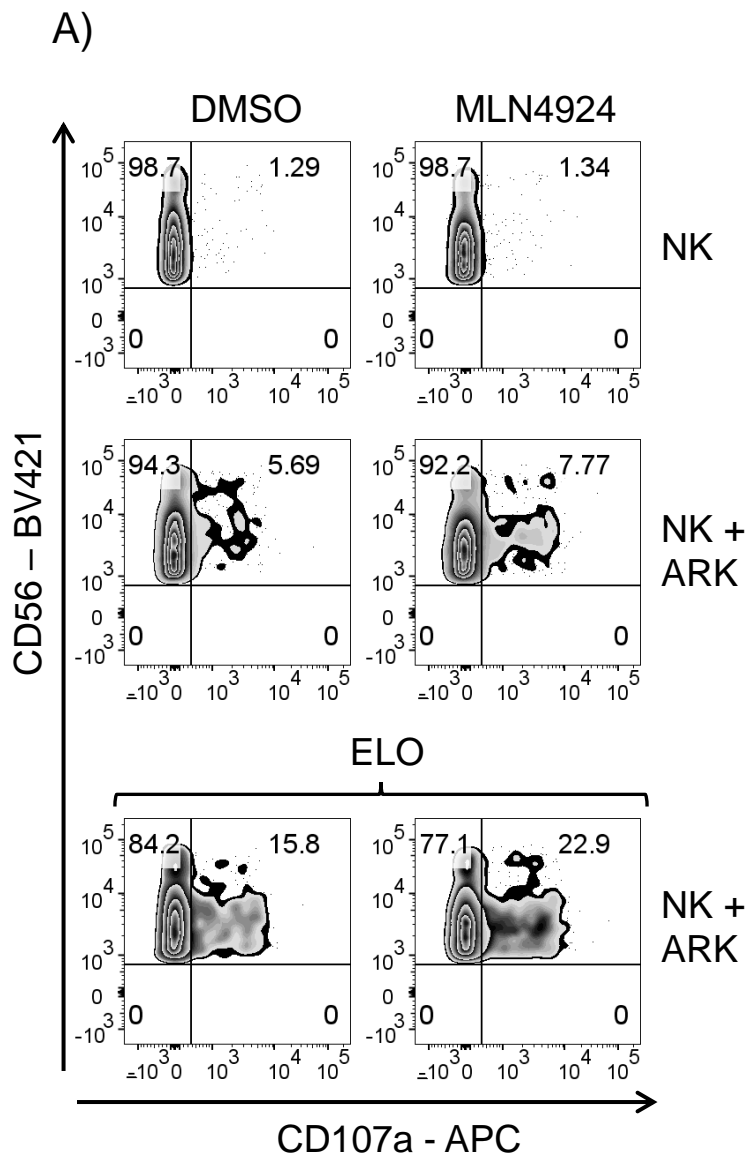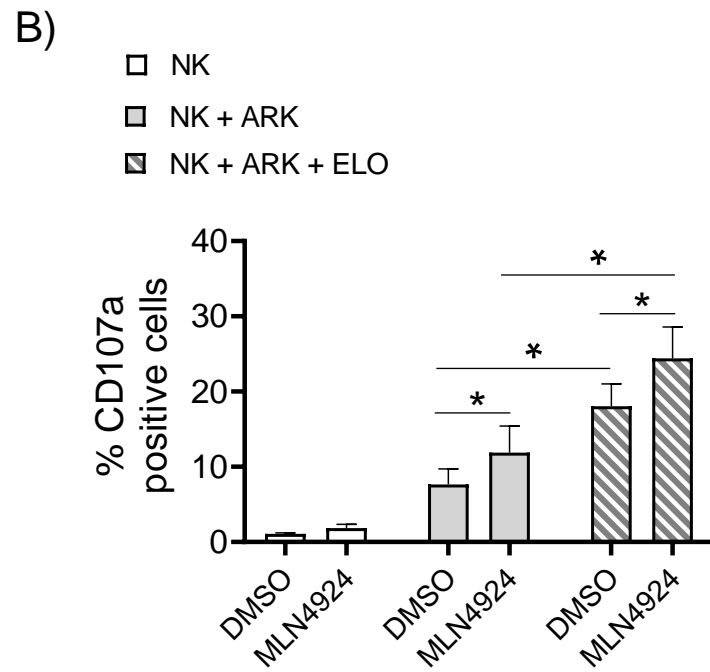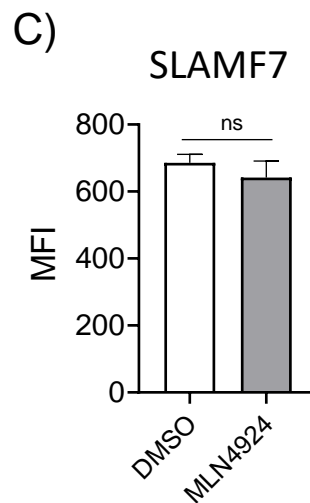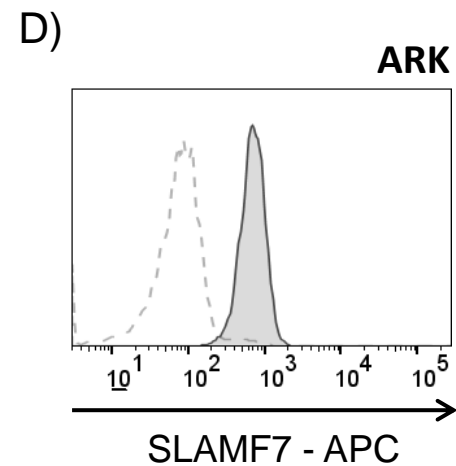

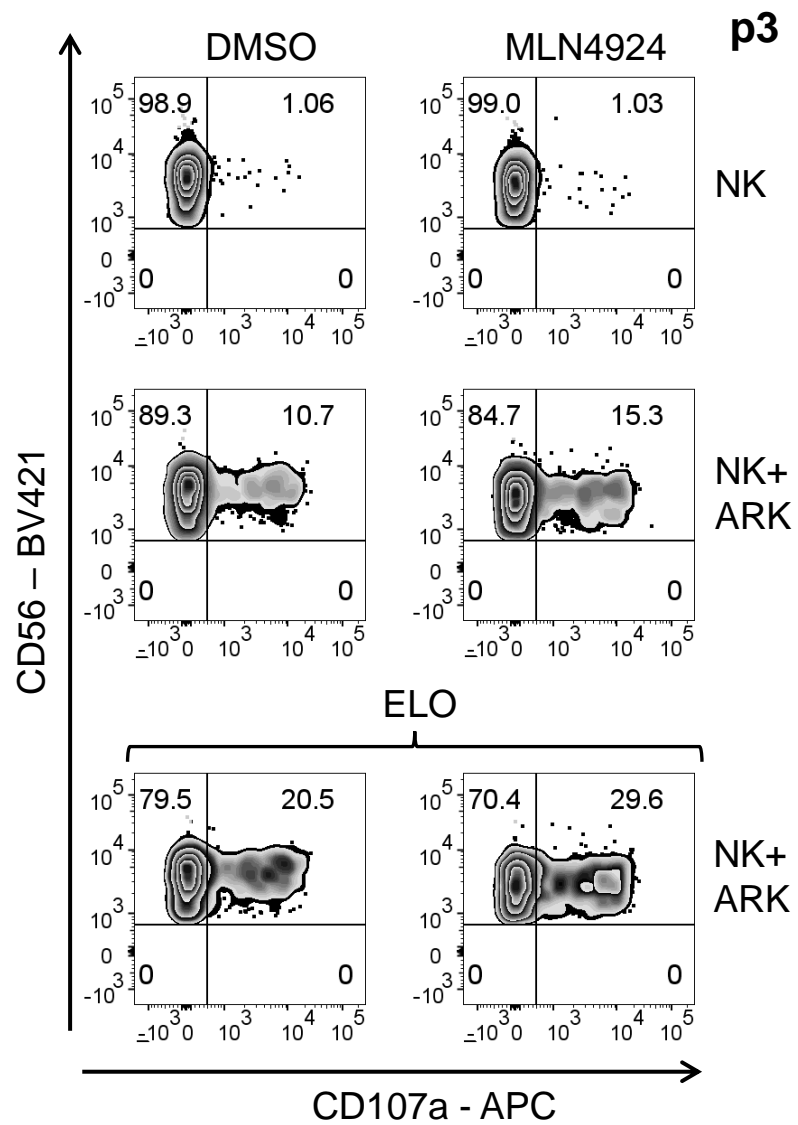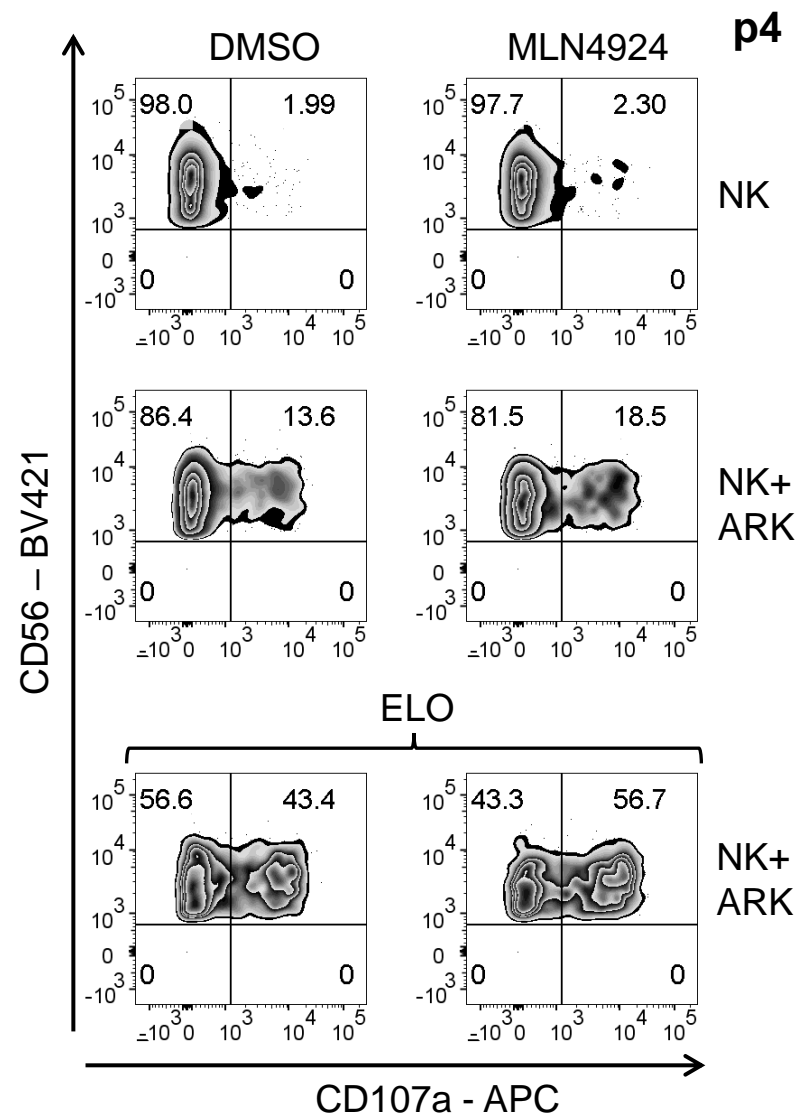

Suppl. Fig. 8

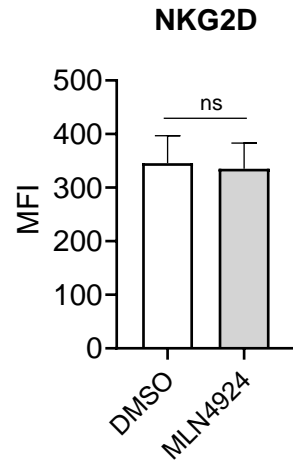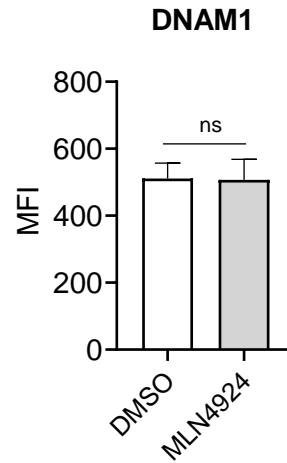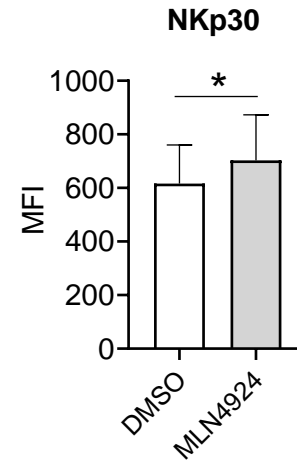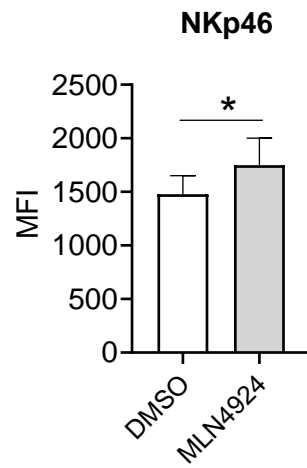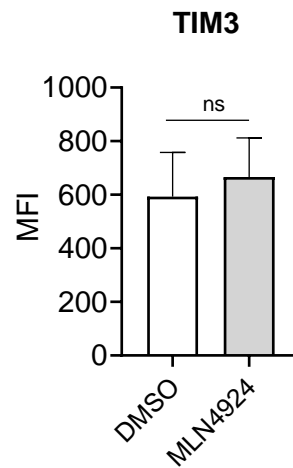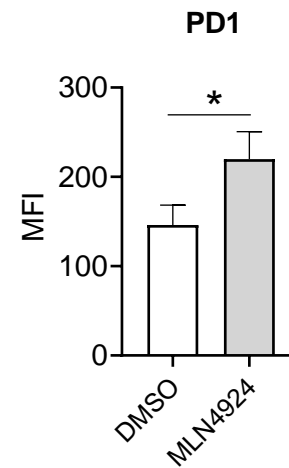

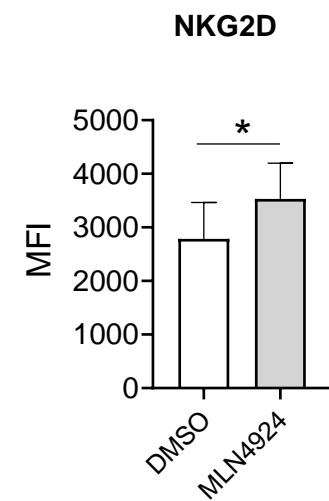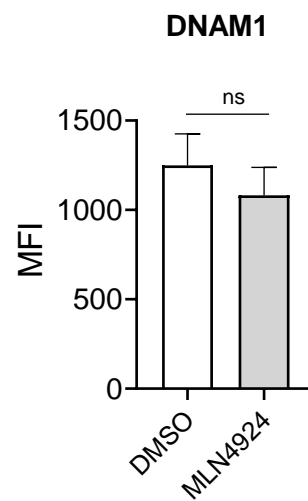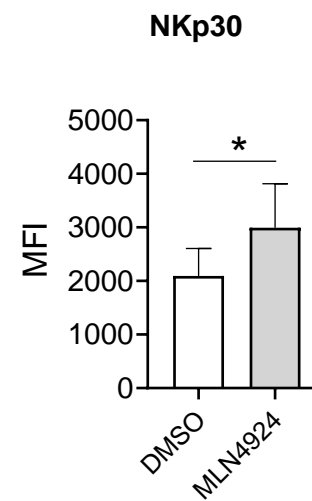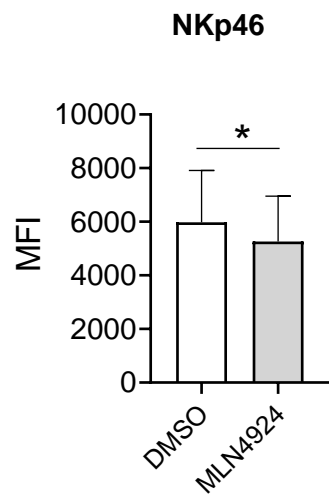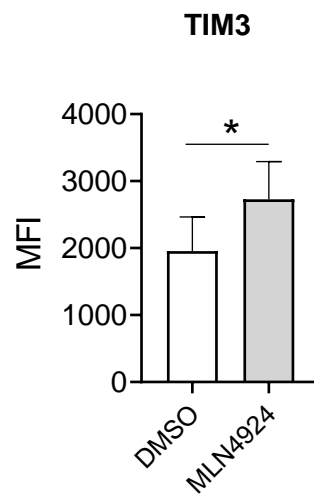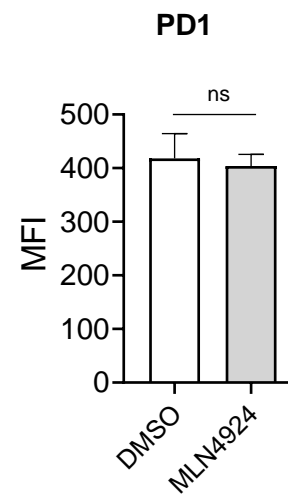

A)

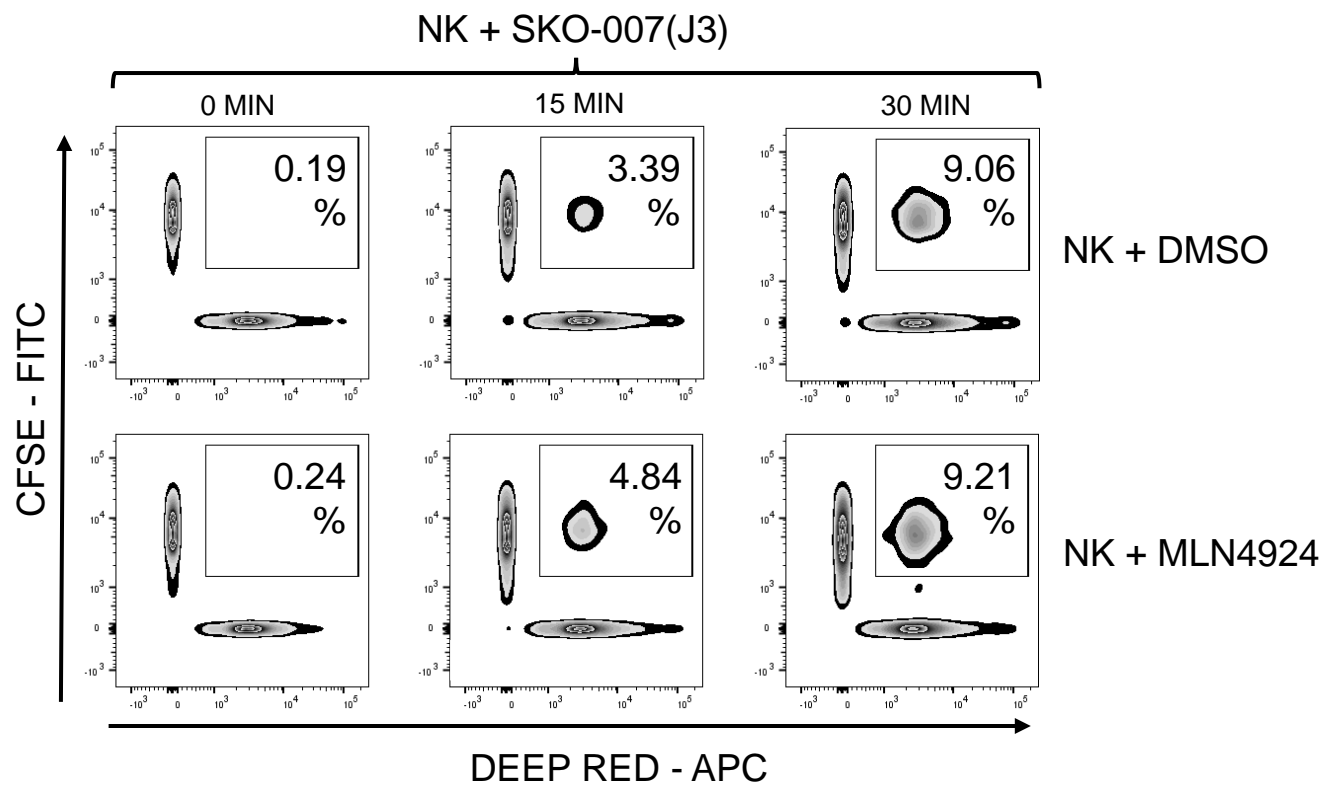

B)

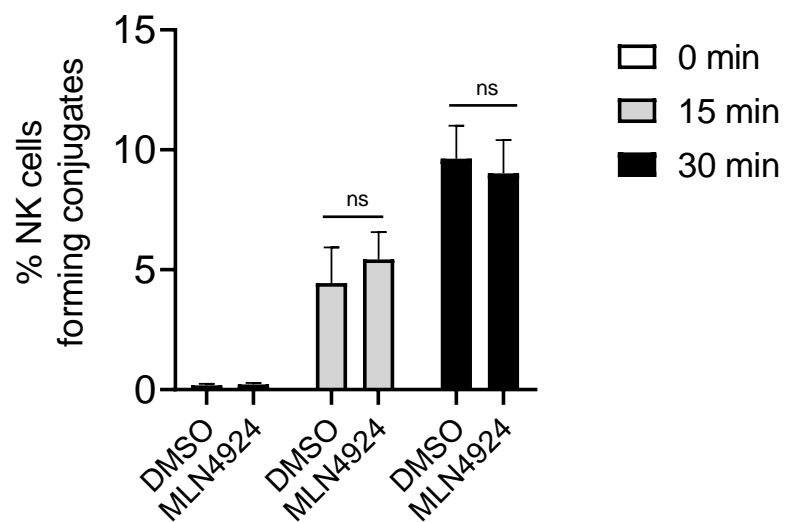

A)

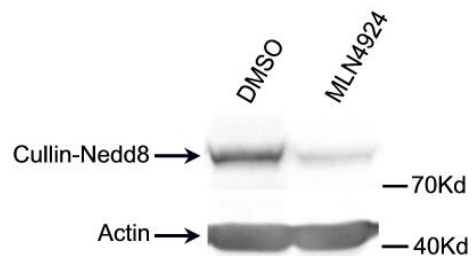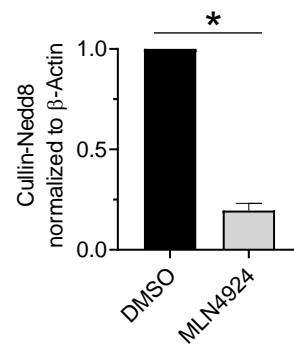

B)

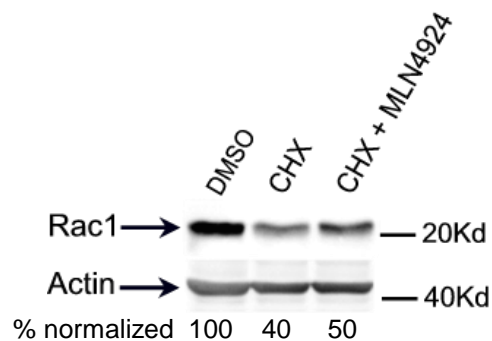

C)

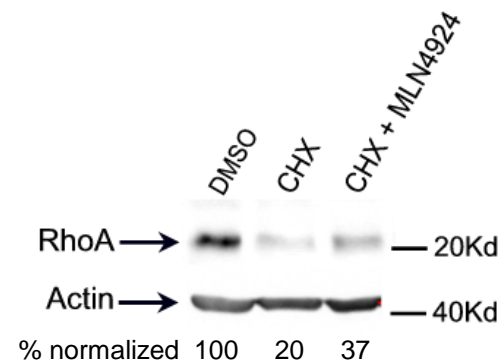

A)

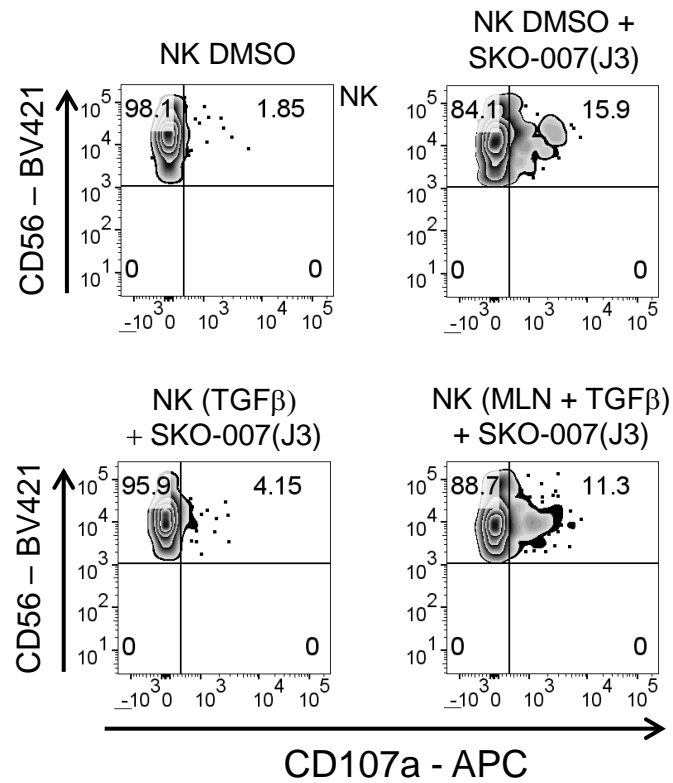

B)

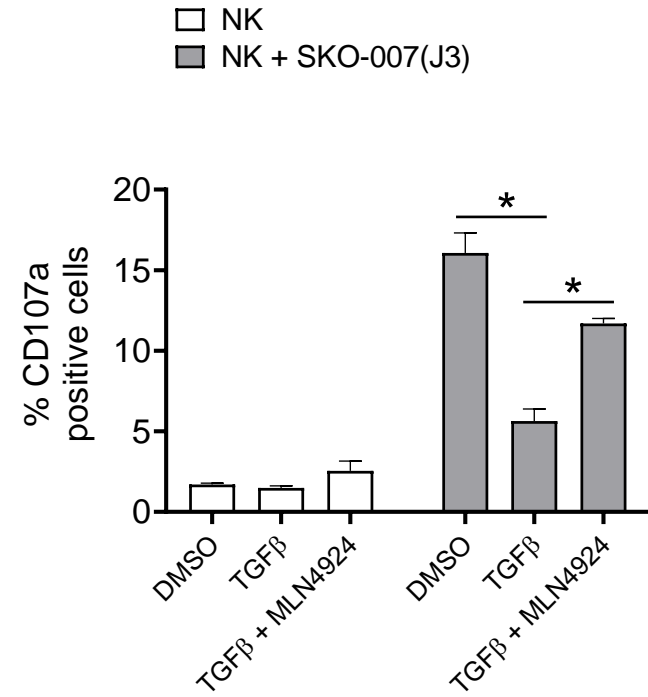

A)

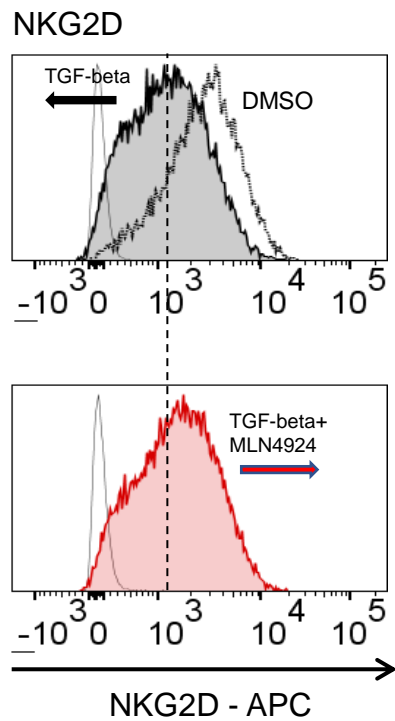

B)

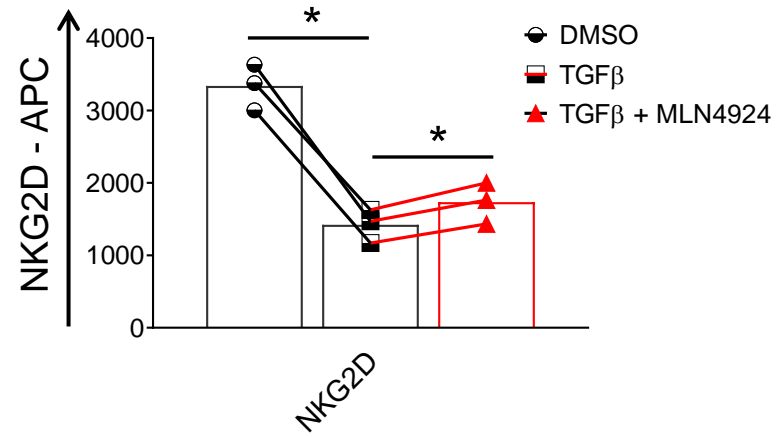

C)

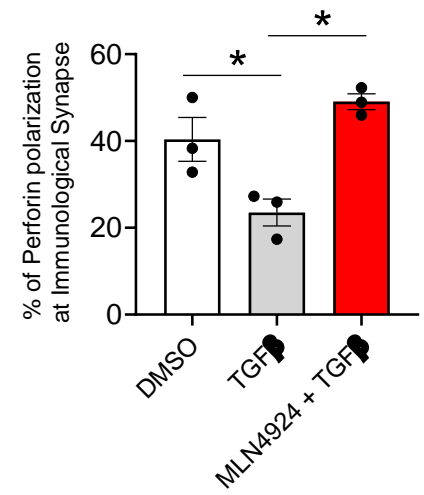

A)

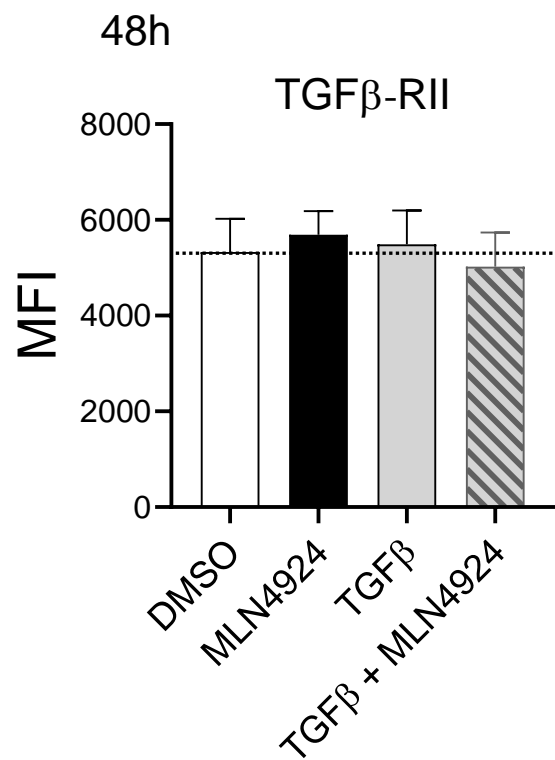

B)

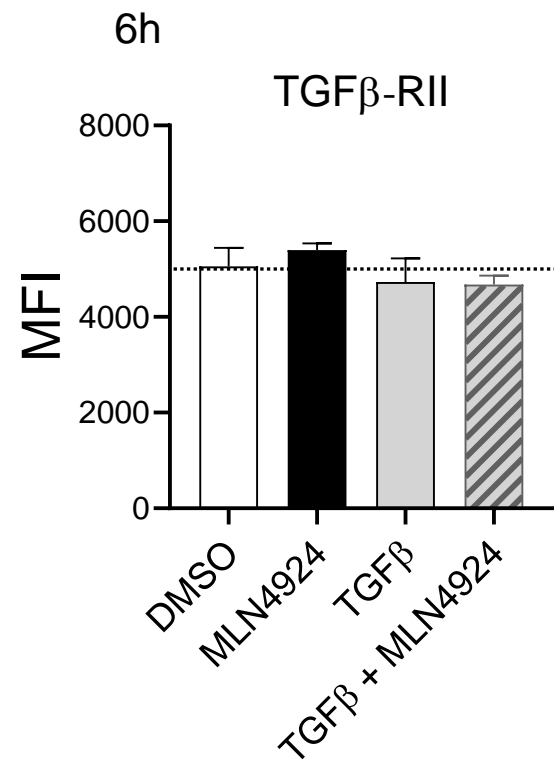

Supplement: Supplementary file 1 — Suppl. Figures [file 41419_2023_5949_MOESM1_ESM.pdf]

Figure 6A

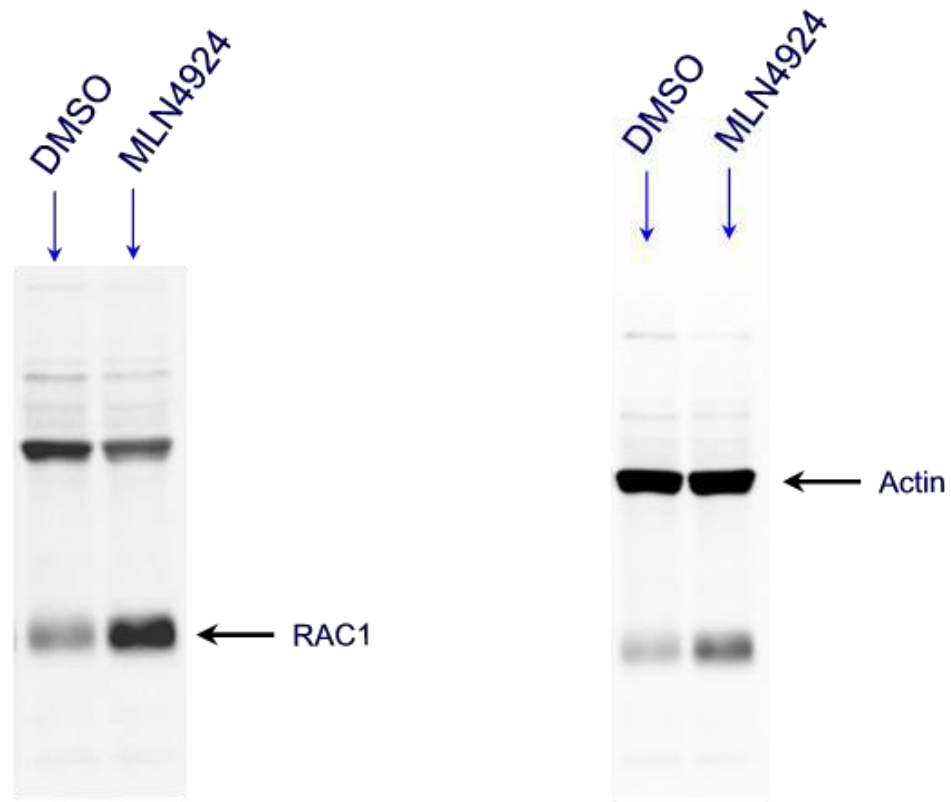

Figure 6B

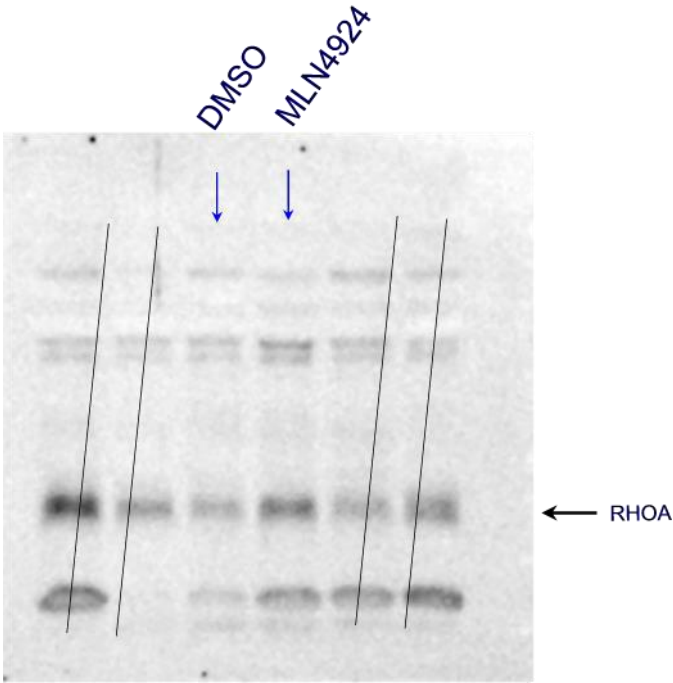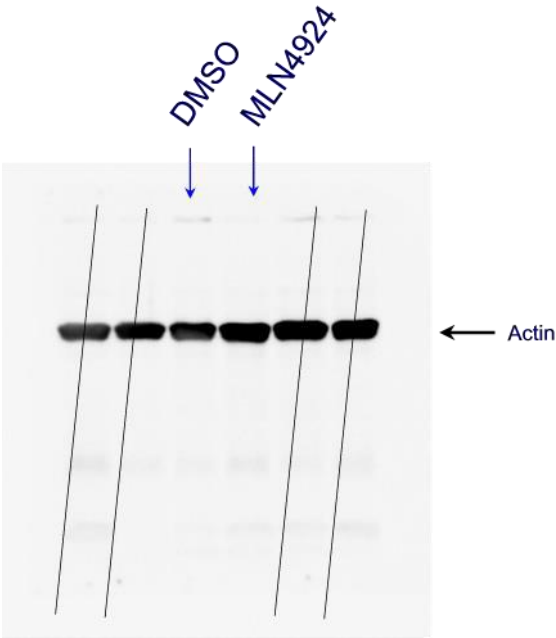

Figure 8C

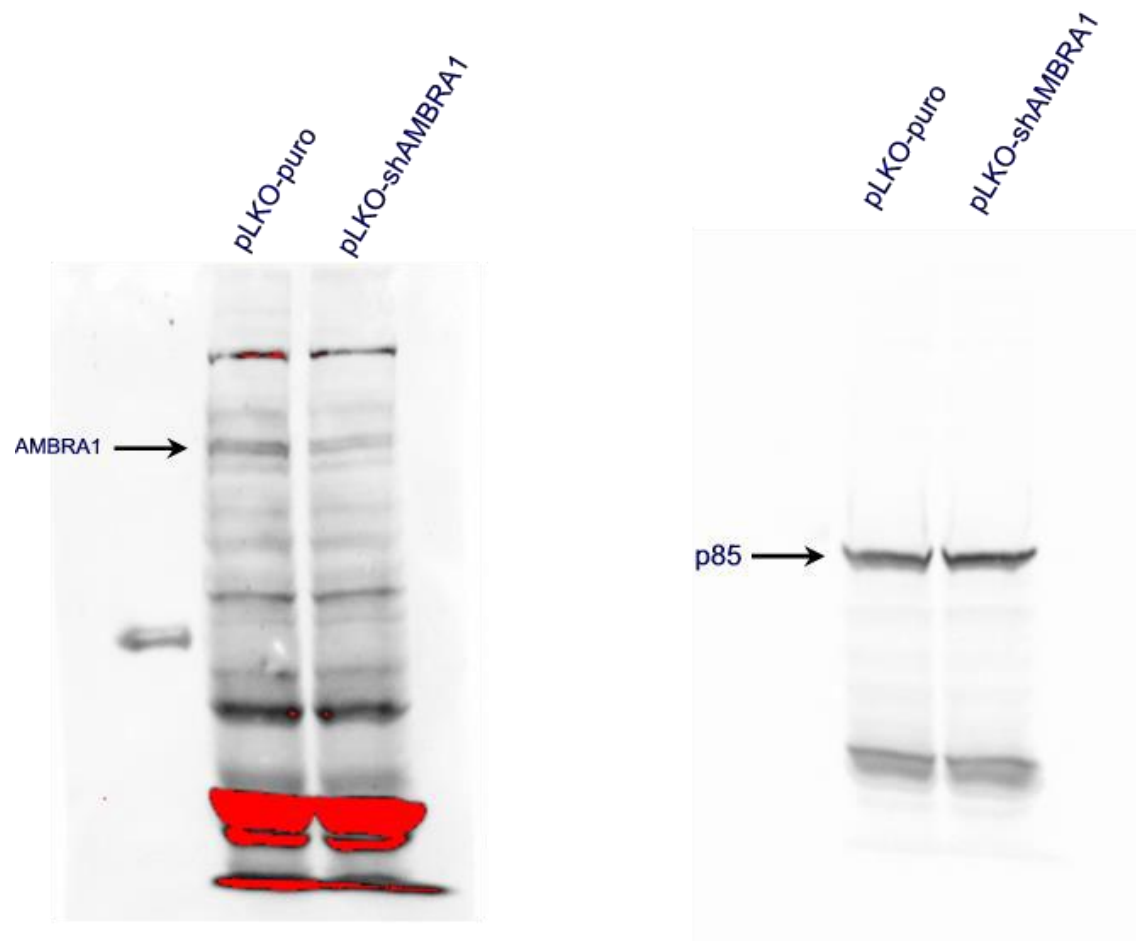

Suppl. Fig. 12A

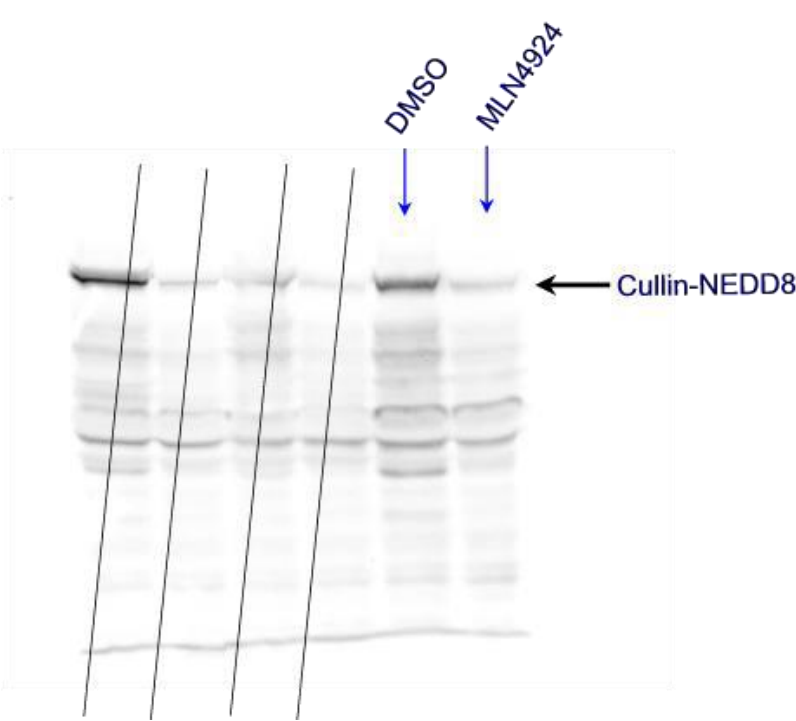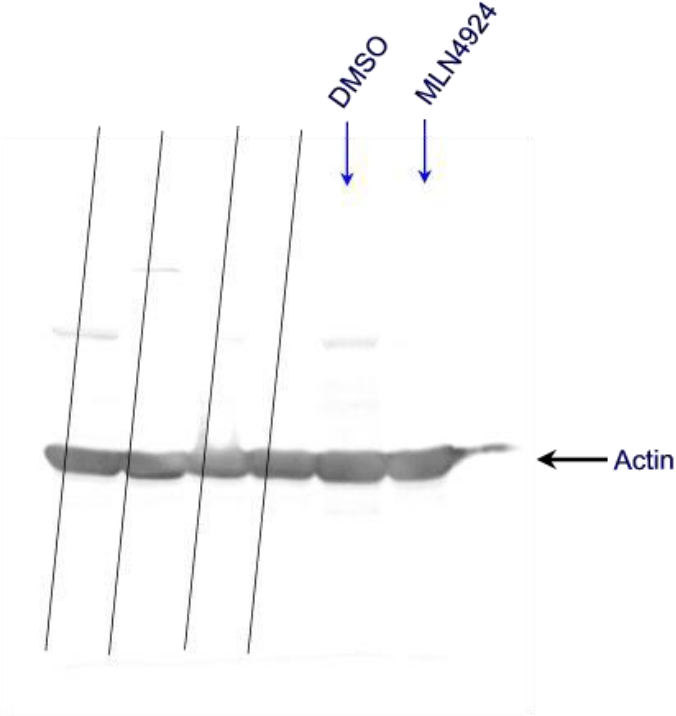

Suppl. Fig. 12B

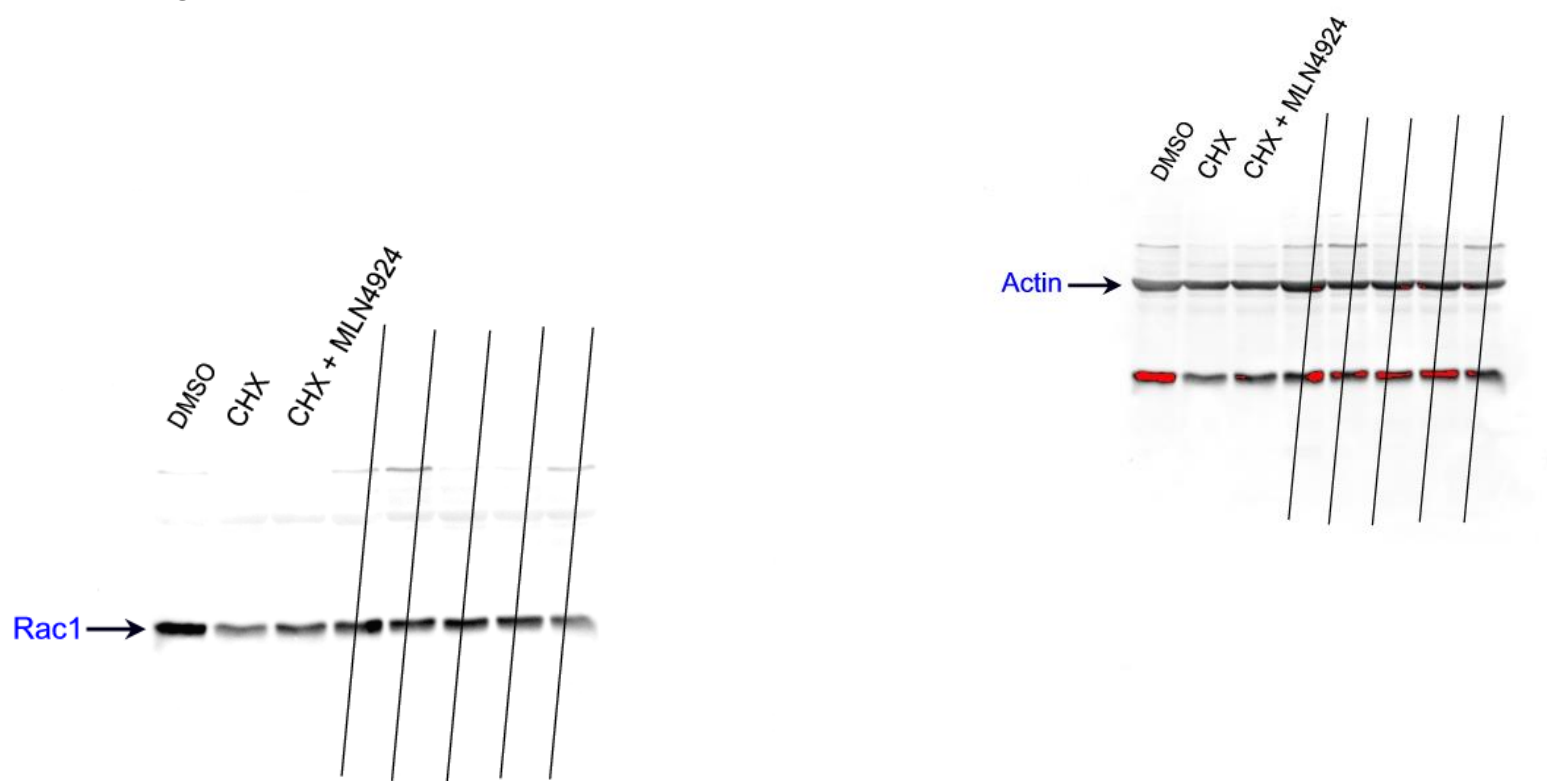

Suppl. Fig. 12C

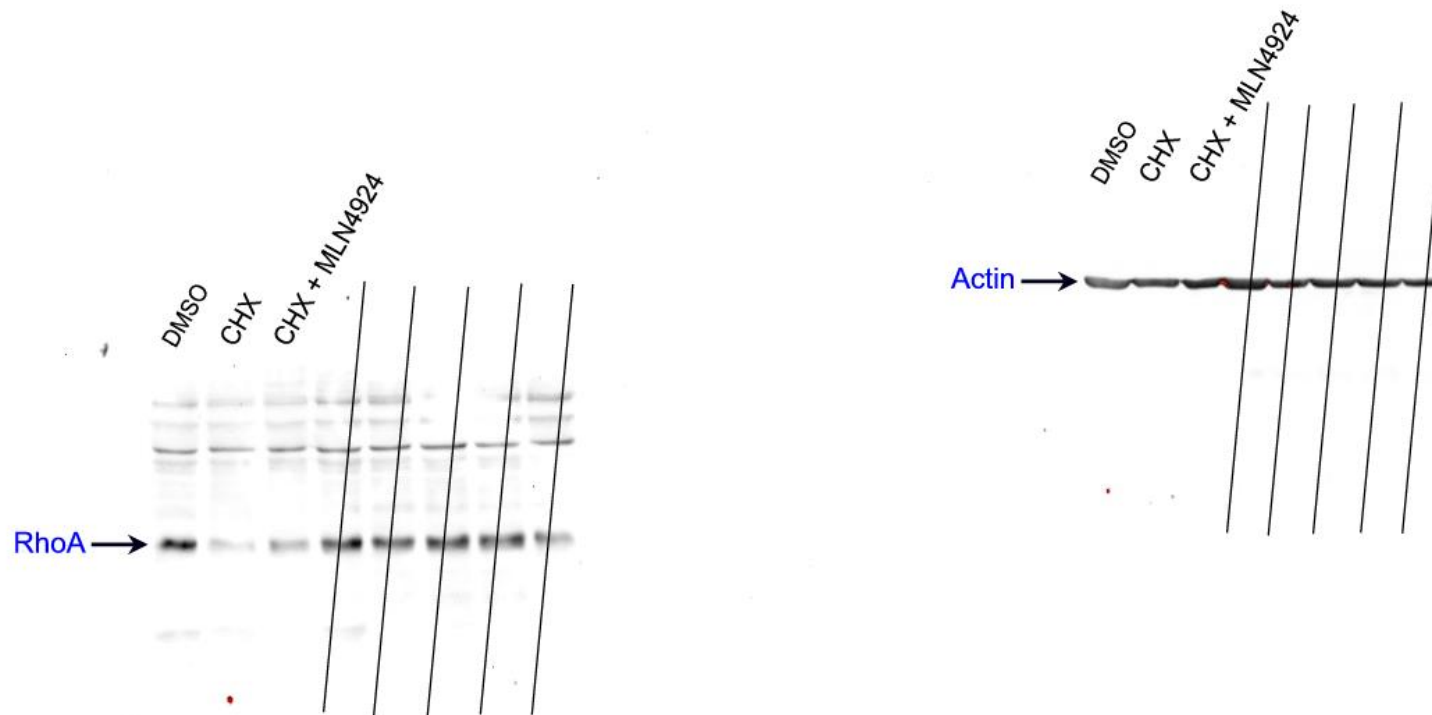

Supplement: Supplementary file 3 — Uncropped WBs [file 41419_2023_5949_MOESM3_ESM.pdf]
