# Supplementary material for: NEDD8-activating enzyme inhibition potentiates the anti-myeloma activity of natural killer cells
Source: Cell Death Dis. 2023 Jul 17;14(7):438. doi: 10.1038/s41419-023-05949-z (PMC10352239; doi:10.1038/s41419-023-05949-z)
Supplement: Supplementary file 2 — Suppl. Fig. Legends [file 41419_2023_5949_MOESM2_ESM.docx]

**NEDD8-activating enzyme inhibition potentiates the anti-Multiple Myeloma activity of Natural Killer cells.**

Sara Petillo^1^, Elena Sproviero^1^, Luisa Loconte^1^, Lorenzo Cuollo^1^, Alessandra Zingoni^1^, Rosa Molfetta^1^, Cinzia Fionda^1^, Alessandra Soriani^1^, Cristina Cerboni^1^, Maria Teresa Petrucci^2^, Francesca Fazio^2^, Rossella Paolini^1^, Angela Santoni^1,3,4^ and Marco Cippitelli^1*^.

**Supplementary figure legends.**

Suppl. Fig. 1 - **Gating strategy to study NK cells out of total PBMCs purified from healthy donors.** CD107a expression was analyzed on CD14^-^CD19^-^CD45^+^CD3^-^CD138^-^CD56^+^ NK cells.

Suppl. Fig. 2 - **MLN4924 increases NK cell degranulation against MM cell lines.** A, B) NK cell-mediated degranulation was evaluated, as described in Fig. 1, using as target cells ARK and RPMI 8226 MM cell lines. The assay was performed at the E/T ratio of 2.5:1 in complete medium at 37°C and 5% CO_2_ for 2 h. Cells were stained with Fixable Viability Stain 780, anti-CD14-APC-H7, anti-CD19-APC-H7, anti-CD45-PE-Cy™7, anti-CD3-BV510, anti-CD138-PE, anti-CD56-BV421, anti-CD16-PerCP-Cy™5.5 and anti-CD107a-APC. CD107a expression was evaluated on NK cells gated as CD14^-^CD19^-^CD45^+^CD3^-^CD138^-^CD56^+^ cells using a FACS Canto II flow cytometer and data were analyzed by FlowJo V10 Cytometric Analysis Software (BD Biosciences). Percentage of CD107a positive cells represents the average of three independent experiments and statistical significance was evaluated by paired Student’s *t* test (**P*< 0.05).

Suppl. Fig. 3 - **Gating strategy to study NK cells out of CD138^-^ cells (BMMCs depleted of MM cells) purified from MM patients.** Cell surface expression of CD107a was analyzed on CD14^-^CD19^-^CD45^+^CD3^-^CD138^-^CD56^+^ NK cells.

Suppl. Fig. 4 - **Patient-derived NK cells treated with MLN4924 show increased degranulation against MM cell lines.** A, B) NK cell-mediated degranulation was evaluated as described in Fig. 2, using as target ARK and RPMI 8226 MM cell lines. The assay was performed at the E/T ratio of 2.5:1 in complete medium at 37°C and 5% CO_2_ for 2 h. Cells were stained with Fixable Viability Stain 780, anti-CD14-APC-H7, anti-CD19-APC-H7, anti-CD45-PE-Cy™7, anti-CD3-BV510, anti-CD138-PE, anti-CD56-BV421, anti-CD16-PerCP-Cy™5.5 and anti-CD107a-APC. CD107a expression was analyzed on CD14^-^CD19^-^CD45^+^CD3^-^CD138^-^CD56^+^ NK cells using a FACS Canto II flow cytometer (BD Biosciences) and data were analyzed by FlowJo V10 Cytometric Analysis Software (BD Biosciences). Percentage of CD107a positive cells was analyzed in three independent experiments and statistical significance was evaluated by paired Student’s *t* test (**P*< 0.05).

Suppl. Fig. 5 - **Reduced expression of NAE increases NK cell degranulation against SKO-007(J3) MM cells.** A) Degranulation of NK92 cells against SKO-007(J3) cells was evaluated using the lysosomal marker CD107a. NK92 cells, infected with NAE1 or UBA3 shRNA-lentivirus (or control lentivirus) were selected for a week with puromycin (1 µg/ml) and then co-cultured with the SKO-007(J3) cell line used as target. The assay was performed at the Effector/Target (E/T) ratio of 2.5:1 in complete medium at 37°C and 5% CO_2_ for 2 h. Cells were then stained with Fixable Viability Stain 780, anti-CD138-PE, anti-CD56-BV421 and anti-CD107a-APC. CD107a expression was evaluated on NK92 cells gated as CD138^-^CD56^+^ using a FACS Canto II flow cytometer (BD Biosciences) and data were analyzed by FlowJo V10 Cytometric Analysis Software (BD Biosciences). A representative experiment is shown. B) Percentage of CD107a positive cells represents the average of two independent experiments. C) Real Time PCR analysis of total mRNA obtained from NK92 cells infected with the indicated lentiviral shRNA vectors. Data, expressed as fold change units, were normalized to GAPDH and referred to the pLKO/control-infected cells considered as calibrator.

Suppl. Fig. 6 - **Patient-derived NK cells treated with MLN4924 show increased Daratumumab-mediated degranulation against MM cells.** NK cell-mediated degranulation was evaluated as described in Fig. 3, using as target cells ARK MM cell line. The assay was performed at the E/T ratio of 2.5:1 in complete medium at 37°C and 5% CO_2_ for 2 h. Cells were stained with Fixable Viability Stain 780, anti-CD14-APC-H7, anti-CD19-APC-H7, anti-CD45-PE-Cy™7, anti-CD3-BV510, anti-CD138-PE, anti-CD56-BV421, anti-CD16-PerCP-Cy™5.5 and anti-CD107a-APC. Fluorescence was analyzed using a FACS Canto II flow cytometer (BD Biosciences) on CD14^-^CD19^-^CD45^+^CD3^-^CD138^-^CD56^+^ NK cells and data were analyzed by FlowJo V10 Cytometric Analysis Software (BD Biosciences). Data obtained from two patients are shown.

Suppl. Fig. 7 - **Elotuzumab-mediated degranulation against the MM cells is increased by Neddylation inhibition.** A) NK cell degranulation was evaluated using CD107a as previously described. As source of effector cells, we used PBMCs stimulated with MLN4924 (150 nM) or vehicle for 72 h and incubated with ARK cells. Anti-SLAMF7/Elotuzumab (ELO) has been added to the co-culture at the final concentration of 1 µg/10^6^ cells, during the incubation of 2h at 37°C and 5% CO_2_. Cells were stained with Fixable Viability Stain 780, anti-CD14-APC-H7, anti-CD19-APC-H7, anti-CD45-PE-Cy™7, anti-CD3-BV510, anti-CD138-PE, anti-CD56-BV421, anti-CD16-PerCP-Cy™5.5 and anti-CD107a-APC, and CD107a expression was analyzed using a FACSCanto II flow cytometer (BD Biosciences) on CD14^-^CD19^-^CD45^+^CD3^-^CD138^-^CD56^+^ NK cells. Data were analyzed by FlowJo V10 Cytometric Analysis Software (BD Biosciences). A representative experiment is shown. B) Average percentage of CD107a positive cells was calculated based on three independent experiments and statistical significance was evaluated by ANOVA (**P*< 0.05). C) Expression of SLAMF7 on NK cells (gated on PBMCs), untreated or treated with MLN4924 (150 nM) for 72 h. Histograms represents the average of four independent experiments. Statistical significance was evaluated by paired Student’s *t*-test (**P* < 0.05). D) Expression of SLAMF7 on ARK MM cells.

Suppl. Fig. 8 - **Patient-derived NK cells treated with MLN4924 show increased Elotuzumab-mediated degranulation against MM cells.** NK cell-mediated degranulation was evaluated as described in Fig. 3, using as target cells ARK MM cell line. The assay was performed at the E/T ratio of 2.5:1 in complete medium at 37°C and 5% CO_2_ for 2 h. Cells were stained with Fixable Viability Stain 780, anti-CD14-APC-H7, anti-CD19-APC-H7, anti-CD45-PE-Cy™7, anti-CD3-BV510, anti-CD138-PE, anti-CD56-BV421, anti-CD16-PerCP-Cy™5.5 and anti-CD107a-APC. Fluorescence was analyzed using a FACS Canto II flow cytometer (BD Biosciences) on CD14^-^CD19^-^CD45^+^CD3^-^CD138^-^CD56^+^ NK cells and data were analyzed by FlowJo V10 Cytometric Analysis Software (BD Biosciences). Data obtained from two patients are shown.

Suppl. Fig. 9 - **Modulation of activating and inhibitory receptors by MLN4924 on NK cells from PBMCs.** Expression of the indicated receptors was evaluated in NK cells gated from PBMCs as described above. PBMCs, untreated or treated MLN4924 (150 nM) for 72 h, were stained with Fixable Viability Stain 780, anti-CD14-APC-H7, anti-CD19-APC-H7, anti-CD45-PE-Cy™7, anti-CD3-BV510, anti-CD56-BV421/PE and mAbs for the indicated activating or inhibitory receptors: anti-NKG2D-APC, anti-DNAM1-FITC, anti-NKp30-Alexa Fluor® 647, anti-NKp46-PE, anti-TIM-3-BB515, anti-PD-1-BV421. Fluorescence was analyzed using a FACSCanto II flow cytometer (BD Biosciences) and data were analyzed by FlowJo V10 Cytometric Analysis Software (BD Biosciences). The indicated average MFI was calculated on five independent experiments and statistical significance was evaluated by paired Student’s *t* test (**P*< 0.05).

Suppl. Fig. 10 - **Modulation of activating and inhibitory receptors by MLN4924 on cultivated NK cells.** Cultivated NK cells from healthy donors, untreated or treated with MLN4924 (150 nM) for 48 h were stained with Fixable Viability Stain 780, anti-CD3-BV510, anti-CD56-BV421/PE and a mAb against the indicated activating or inhibitory receptors: anti-NKG2D-APC, anti-DNAM1-FITC, anti-NKp30-Alexa Fluor® 647, anti-NKp46-PE, anti-TIM-3-BB515, anti-PD-1-BV421. Fluorescence was analyzed using a FACS Canto II flow cytometer (BD Biosciences) and data were analyzed by FlowJo V10 Cytometric Analysis Software (BD Biosciences). The indicated MFI average value for each receptor was calculated on the basis of at least three independent experiments and statistical significance was evaluated by paired Student’s *t* test (**P*< 0.05).

Suppl. Fig. 11 - **Neddylation inhibition does not affect NK-MM cell conjugate formation.** Conjugate formation between CFSE-labeled cultivated NK cells and Cell Tracker DEEP RED-loaded SKO-007(J3) cells was not affected by a 48 h-treatment with MLN4924 (150 nM), at different time points. A) A representative experiment is shown. Double-positive cells are intended as conjugates. B) Histograms representing the average percentage of NK cells forming conjugates, determined as NK cells bound to SKO-007(J3) target cells out of the total NK cells. The mean of three independent experiments is represented and statistical significance was evaluated by ANOVA.

Suppl. Fig. 12 - **Neddylated-Cullin, Rac1 and RhoA expression in cultivated NK cells exposed to MLN4924.** A) Western blot analysis of Neddylated-Cullin in cultivated NK cells untreated or treated with MLN4924 (150 nM) for 48 h. A representative blot with densitometric analysis is shown. B, C) NK cells were treated with 10 µg/ml CHX or CHX + MLN4924 (150 nM) for 24 h and then collected for Rac1 and RhoA western blot analysis. Representative blots for Rac1 and RhoA with densitometric analysis, of two experiments are shown.

Suppl. Fig. 13 - **Inhibition of NK cell degranulation by TGFβ is partially reverted by MLN4924.** A) NK cell degranulation was evaluated using the lysosomal marker CD107a as previously described. As source of effector cells, we used PBMCs from healthy donors, untreated or treated with recombinant TGFβ (10 ng/ml) and, in some cases, together with MLN4924 (150 nM) for 48h. After incubation, cells were co-cultured with SKO-007(J3) cells used as targets. The assay was performed at the E/T ratio of 2.5/1 in complete medium at 37°C and 5% CO_2_ for 2 h. Cells were stained with Fixable Viability Stain 780, anti-CD14-APC-H7, anti-CD19-APC-H7, anti-CD45-PE-Cy™7, anti-CD3-BV510, anti-CD56-BV421, anti-CD16-PerCP-Cy™5.5 and anti-CD107a-APC. Cell surface expression of CD107a was analyzed on CD14^-^CD19^-^CD45^+^CD3^-^CD56^+^ cells using a FACS Canto flow cytometer (BD Biosciences) and data were analyzed by FlowJo V10 Cytometric Analysis Software (BD Biosciences). A representative experiment is shown. B) Average percentage of CD107a positive cells was calculated based on three independent experiments. Statistical significance was evaluated by ANOVA (**P*< 0.05).

Suppl. Fig. 14 - **Lower expression of NKG2D in cultivated NK cells conditioned with TGFβ is partially reverted by MLN4924.** NKG2D expression on cultivated NK cells (CD3^-^CD56^+^) was evaluated by flow cytometry after a 48h treatment with TGFβ 10ng/ml ± MLN4924 (150 nM). A) a representative experiment is shown, with gray filled histograms indicating the expression of the receptor in the presence of TGFβ, and red filled histograms indicating the expression of the receptor in the presence of TGFβ + MLN4924. B) The MFI values of NKG2D, calculated based on three independent experiments, are shown. Statistical significance was evaluated by ANOVA (**P*< 0.05). C) Perforin polarization at the IS has been analyzed by confocal microscopy in cultivated NK cells, untreated or treated with TGFβ 10ng/ml ± MLN4924 (150 nM) for 48 hours and contacting SKO-007(J3) cells for 15 min. Cells were fixed with paraformaldehyde and after permeabilization were stained for Perforin, F-Actin/Phalloidin and the nuclei were stained with DAPI as described in Material and Methods. Histograms represent the mean (± SEM) of the % of perforin polarization at IS analyzed on at least 50 conjugates present in randomly acquired fields, from three independent experiments (***P*<0.05) (F).

Suppl. Fig. 15 - **Neddylation inhibition does not modulate the expression of TGFβ-RII on NK cells.** Expression of TGFβ-RII was evaluated in NK cells gated on PBMCs from healthy donors. PBMCs, untreated or treated with MLN4924 (150 nM), TGFβ (10 ng/ml) or both for 48h (A) and 6h (B), were stained with Fixable Viability Stain 780, anti-CD14-APC-H7, anti-CD19-APC-H7, anti-CD45-PE-Cy™7, anti-CD3-BV510, anti-CD56-BV421, anti-CD16-PerCP-Cy™5.5 and mAb against TGFβ-RII-APC. Fluorescence was analyzed on CD14^-^CD19^-^CD45^+^CD3^-^CD56^+^ NK cells using a FACS Canto flow cytometer (BD Biosciences), and data were analyzed by FlowJo V10 Cytometric Analysis Software (BD Biosciences). The indicated MFI values were calculated based on three independent experiments and no statistically significant differences were observed by paired Student’s *t* test.
